# Microalgae-based bacteria for oral treatment of ASD through enhanced intestinal colonization and homeostasis

**DOI:** 10.7150/thno.103737

**Published:** 2025-01-13

**Authors:** Rongrong Yang, Li Ma, Huan Peng, Yifang Zhai, Gengyao Zhou, Linjuan Zhang, Lixia Zhuo, Wei Wu, Yuxi Guo, Jiao Han, Linlin Jing, Xinyu Zhou, Xiancang Ma, Yan Li

**Affiliations:** 1 Department of Psychiatry, The First Affiliated Hospital of Xi'an Jiaotong University, Xi'an, 710061, China.; 2 Center for Brain Science, The First Affiliated Hospital of Xi'an Jiaotong University, Xi'an, 710061, China.; 3 Department of Anesthesiology and Perioperative Medicine, The First Affiliated Hospital of Xi'an Jiaotong University, Xi'an, 710061, China.; 4 Department of Pharmacy, the First Affiliated Hospital of Xi'an Jiaotong University, Xi'an, 710061, China.; 5 Department of Psychiatry, The First Affiliated Hospital of Chongqing Medical University, 400016, Chongqing, China.; 6 Shaanxi Belt and Road Joint Laboratory of Precision Medicine in Psychiatry, The First Affiliated Hospital of Xi'an Jiaotong University, Xi'an, 710061, China.; 7 Shaanxi Provincial Key Laboratory of Biological Psychiatry, The First Affiliated Hospital of Xi'an Jiaotong University, Xi'an, 710061, China.

**Keywords:** ASD, intestinal bacteria, colonization, *Spirulina platensis*, therapeutic effects

## Abstract

**Rationale:** Exogenous supplementation of beneficial intestinal bacteria can alleviate the behavioural symptoms of psychiatric disorders, such as autism spectrum disorder (ASD), through gut-brain interactions. However, the application of beneficial bacteria, such as *Lactobacillus reuteri* (*L. reuteri*), for treating ASD is hindered by limited gut colonization.

**Methods:** Utilizing* Spirulina platensis* (SP) as a natural microcarrier for intestinal bacteria, a safer and more natural binding approach was employed to bind intestinal bacteria to the surface of SP to produce SP-intestinal bacteria. Due to the high adhesion efficiency and long residence time of SP in the intestines, SP-intestinal bacteria exhibit stronger intestinal colonization ability and better therapeutic effects on ASD.

**Results:** SP is an effective carrier that can bind and deliver bacteria of different shapes and with different gram-staining properties. SP-intestinal bacteria exhibited enhanced colonization capabilities both *ex vivo* and *in vivo*. Further research showed that SP-*L. reuteri* had greater intestinal colonization efficiency than *L. reuteri*. SP-*L. reuteri* showed a stronger therapeutic effect on alleviating social deficits in an ASD mouse model by modulating the gut microbiota, enhancing intestinal barrier integrity, reducing lipopolysaccharide entry into the blood and mediating the neuroinflammatory response in the paraventricular thalamic nucleus.

**Conclusions:** This study reports a microalgae-assisted intestinal bacterial delivery system for enhancing intestinal bacterial transplantation for gut-brain axis- or other intestinal-related diseases.

## Introduction

Dysbiosis or imbalance in the gut microbial community has been associated with several mental illnesses, including autism spectrum disorder (ASD) and attention-deficit hyperactivity disorder 1, 2. ASD is a heterogeneous neurodevelopmental disorder characterized by social deficits and repetitive behaviours 3. Individuals with ASD often suffer from various gastrointestinal problems 4, which are associated with changes in the gut microbiota. Therefore, many intervention strategies are designed to alleviate the clinical symptoms of ASD through the modulation of gut-brain communication with probiotic supplements. Precise microbial treatments, such as those based on *Lactobacillus reuteri* (*L. reuteri*), selectively rescue social deficits 5, 6. Supplementation with probiotics has emerged as a promising therapeutic intervention for ASD treatment.

However, exogenous beneficial gut bacteria exhibit a short retention time in the intestine and a low colonization efficiency 7 due to the inherently hostile gastrointestinal environment, such as gastrointestinal transit and gastric acid. A variety of strategies have been explored to improve the intestinal colonization efficiency of probiotics 8, 9. However, these approaches still yield insufficient probiotic activity, require complex preparation steps, and result in insufficient contact between beneficial bacteria and the intestinal mucosa 10, 11. Therefore, there is an urgent need to develop safer, more efficient, and simpler strategies to enhance the efficiency of intestinal colonization by gut bacteria.

Microalgae are a type of microorganisms rich in nutrients. With the development of technology, the combination of microalgae and biomaterials can improve the therapeutic effect and has attracted increasing attention 12. *Spirulina platensis* (SP) is an edible natural microalga with a length of 200-500 μm and a three-dimensional spiral structure. SP has many biological activities, such as anti-inflammatory effects and regulation of the intestinal flora balance, and helps to prevent and treat a variety of intestinal diseases 13, 14 and mental diseases 15. The length, helical structure, and large surface area of SP increase its likelihood of being trapped by the intestinal villi, notably prolonging the retention time of SP in the host intestine 16. Therefore, leveraging the exceptional properties of microalgae for drug delivery and optimizing their qualities is of paramount importance 17. The combined use of SP with small molecule drugs and biological materials also has significant therapeutic effects on intestinal 14, 16 and mental diseases 18.

Bacteria adhere to other biological surfaces through adsorption and subsequent formation of microcolonies and biofilms 19, 20. Based on the above features of SP, we developed a new system for the SP-assisted delivery of intestinal bacteria using SP as a natural microcarrier. We screened for safer and natural binding pathways to attach intestinal bacteria to the surface of SP to form SP-intestinal bacteria. We evaluated the universality, safety, and intestinal colonization efficiency of different types of SP-intestinal bacteria. To investigate the effectiveness of this delivery system, we compared the therapeutic effects of SP-intestinal bacteria and intestinal bacteria on symptoms in an ASD mouse model. The development of novel oral delivery systems has revealed new approaches for the clinical translation and application of microbial therapy and provided new therapeutic strategies for the treatment of gut-brain axis-mediated psychiatric disorders.

## Materials and methods

### Materials

The various media and components used to culture the bacterial strains were as follows: De Man, Rogosa, and Sharpe media (MRS; Beijing Aobox Technology Co., Ltd.); lysogeny broth (LB; Beijing Aobox Biotechnology Co., Ltd.); fluid thioglycollate media (FT; Hope Biotechnology Co., Ltd.); and ampicillin (amp; Solarbio Life Sciences). SGF and SIF were prepared according to the United States Pharmacopoeia. Briefly, SGF was prepared by dissolving 3.2 g of pepsin and 2.0 g of sodium chloride in 1 L of deionized water, after which the pH was adjusted to 2 with HCl. SIF was generated by dissolving 6.8 g of KH_2_PO_4_ and 10 g of trypsin in 1 L of deionized water and adjusting the pH to 6.8 with NaOH. SP was purchased from Zhejiang Zaoshang Biotechnology Co., Ltd., and cultured in Zarrouk medium (Shanghai Guangyu Biological Technology Co., Ltd.) under conditions of 5000 lux light intensity and a 14/10 h light - dark cycle at 28 °C. All other reagents were purchased from domestic suppliers and used as received.

### Animals

C57BL/6J and BTBR T+ Itpr3tf/J (BTBR) mice were obtained from Xian Huaren Biotechnology Co., Ltd. All of the animal procedures complied with the guidelines of the First Affiliated Hospital of Xi'an Jiaotong University (XJTUAE2023-573). The animals were kept in specific pathogen-free rooms in a temperature- and light-controlled environment. The animals were fed a standard chow diet ad libitum and had free access to water.

### Bacterial strain culture

*L. reuteri* CICC 6123 (*L. reuteri*) and *S. thermophilus* CICC 6260 (*S. thermophilus*) were purchased from the China Center of Industrial Culture Collection (CICC, China). *L. reuteri* and *S. thermophilus* were kept on MRS agar plates and cultured in MRS liquid medium overnight (static culture, 37 °C) before each experiment. *Escherichia coli-*pTurboGFP-B (*E. coli*) was purchased from Miaoling Biology. *E. coli* was kept on an LB agar plate and recultured in LB liquid medium supplemented with amp (1 µg/µL) overnight (220 rpm, 37 °C). *Bacteroides ovatus* ATCC 8483 (*B. ovatus*) was obtained from the BeNa Culture Collection (Beijing, China) and cultured on an LB agar plate.

### Preparation of SP-intestinal bacteria

To establish SP-intestinal bacteria, the obtained clear SP was added to the bacterial culture solution for coculture. An orthogonal rotation combination test design comprising four levels and four factors, namely, rotation speed, OD_600_, SP concentration, and time, was used to optimize the reaction conditions (Table S1). After coculture, the coculture mixture was centrifuged at 900 rpm for 2 min, and the pellet was washed twice with sterile water to remove as many nonloaded bacteria as possible. To determine the number of loaded bacteria, the supernatant obtained after loading the bacteria was collected, uniformly spread onto an LB agar plate and incubated overnight at 37 °C. The resulting bacterial colonies were counted to determine the number of unloaded bacteria. The number of loaded bacteria was then calculated using the following equation: (CFU of bacteria before loading - CFU of unloaded bacteria).

### Physical characterization

The SP-intestinal bacteria were stained using a Gram Stain Tissue Kit (R40080; Thermo Fisher Scientific, China) and imaged with a light microscope (Olympus Ix73, Japan). SP emits red light under 605 nm excitation light, and *E. coli* tagged with GFP emits green light under 495 nm excitation light. Therefore, fluorescence microscopy (Olympus Ix73, Japan) was used to identify the SP-intestinal bacteria. SP-intestinal bacteria were further visualized by scanning electron microscopy (SEM; TESCAN VEGA 3 SBH, Czech Republic). When pre-treatment electron microscope samples, it is essential to handle these samples swiftly and delicately to minimize any damage. The samples were first fixed with cold Karnovsky's fixative overnight, dehydrated in a series of ethanol washes (0%, 50%, 75%, 100%, and 100%), critical point-dried, coated with a thin layer of AuPd and imaged at an accelerating voltage of 10 kV.

### *In vitro* resistance assessment

SP-intestinal bacteria (10^8^ CFU/mL) were immersed in SGF (15, 30, or 60 min) or SIF without or with bile salts (0.3 mg/mL; 1, 2, 3, 4 or 5 h). At different time points after incubation at 37 °C with gentle shaking, the reaction mixture was washed three times with cold phosphate-buffered saline (PBS) and then resuspended in 500 μL of PBS for bacterial plate counting. After 15 min of SGF incubation, SEM was performed to analyse the morphological changes in the bacteria. Unmodified bacteria were used as a control.

### Evaluation of growth capacity after treatment with simulated gastrointestinal fluids

The growth capacity was evaluated according to the growth curve. After the SP-intestinal bacteria had incubated in SGF, SIF, and bile salts for 1 h, the cells were washed twice with sterile PBS and resuspended in PBS. The resuspended samples were inoculated into a 96-well plate with 10^6^ CFU/well in medium. The plate was incubated at 37 °C, and the absorbance at 600 nm (OD_600_) was monitored every hour for 8 or 10 h utilizing a microplate reader (Cytation 5M, Bio-Tek, USA). GraphPad Prism 9.0.1 (GraphPad Software, Inc.) was used to construct growth curves.

### Detection of bioadhesion ability *in vitro*

The experimental protocol was based on previously reported methods 21, with several modifications. The fresh intestinal tissue was divided into sections of approximately 1-2 cm and rinsed three times with PBS to completely remove the intestinal contents, and ligate one end. The prepared stomach and intestinal segments from different locations (duodenum, jejunum, ileum, and colon) were randomly divided into two groups (n = 6 for each group). After adding equal amounts (150 µL) of *E. coli* and SP-*E. coli*, the other end was tightly tied, put into PBS, and shaken up and down slightly (10 rpm/min) for 30 min. All the above operations were performed with the sterilized surgical instruments on a sterile, clean bench. At the designated time points, the intestinal segments were gently rinsed 3 times with PBS to completely eliminate any unattached bacteria. Images were taken to observe the adhesion of SP-*E. coli*, and the intestinal segments were subsequently analysed with an *in vivo* animal fluorescence imaging system (Tanon ABL X6, China), homogenized in PBS, and plated on selective LB plates for counting. Intestinal segments were placed flat on glass slides, and fluorescence scans of 50 μm thickness were acquired using a confocal microscope (Nikon A1+, Xi'an Qian Xin Instrument Co., Ltd., Japan). The adhesion of SP-*E. coli* to intestinal villi was observed via fluorescence microscopy after cryosectioning (NX50, Thermo Fisher Scientific, China). The intestinal segments were subjected to a series of processes, such as fixation, dehydration, and air-drying, before SEM imaging was performed.

For analysis of SP-*L. reuteri* adhesion *in vitro*, we labelled *L. reuteri* with 1,1'-dioctadecyl-3,3,3',3'-tetramethylindotricarbocyanine iodide (DIR), and then, SP-*L. reuteri* (labelled with DIR) was prepared. The experimental procedure was the same as that described above, but the detection indicators used were different. After processing, the intestinal segments were analysed with an animal fluorescence imaging system, homogenized in PBS, and plated on selective MRS plates for counting. The intestinal segments were embedded in paraffin, sectioned, and Gram-stained (G1065-20ML, Servicebio, China) 8. The intestinal segments were subjected to a series of processes, such as fixation, dehydration, and air-drying, before SEM imaging was performed.

### Exploration of intestinal retention in mice

To further evaluate the colonization and retention ability of SP-*E. coli* or SP-*L. reuteri* in the intestine of C57BL/6J mice (male, 6 weeks old), SP-*E. coli* or SP-*L. reuteri* was prepared after labelling the bacteria with DIR dye. The mice were randomly divided into two groups (n = 6), after which the abdominal hair was removed. Fluorescence imaging of the mice was performed via an animal fluorescence imaging system at 2, 4, 6, 8, 10, 16, 24, and 32 h after gavage with equal amounts of *E. coli* and SP-*E. coli* or *L. reuteri* and SP-*L. reuteri*. Gastrointestinal tract tissue was isolated to observe the fluorescence distribution.

The mice (C57BL/6J, male, 4 weeks old) were divided into 2 groups (n = 6) for intragastric administration of *E. coli*, SP-*E. coli*, *L. reuteri*, or SP-*L. reuteri* (10^8^ CFU, 10 mg/kg/day) for 3 days. Faeces were collected, and the mice were sacrificed. Intestinal tissues such as the jejunum, colon, and ileum were collected, and the contents of some tissues were removed. Faeces or tissues were homogenized and diluted with 1 mL of PBS. The homogenate (40 µL) was spread on LB or MRS agar media overnight and cultured at 37 °C, followed by counting and analysis. In addition, *L. reuteri* is a gram-positive bacterium, so the intestinal tissue was also paraffin-embedded and sectioned, followed by Gram staining and observation via a light microscope. *E. coli* emits a green fluorescence signal. Therefore, after the intestinal tissue was cleared of its contents, the fluorescence signal at a thickness of 30 µm was detected using a confocal microscope.

### Biosafety assessment

Six-week-old C57BL/6J mice were randomly divided into 4 groups (n = 6): the control, SP, *L. reuteri*, and SP-*L. reuteri* groups. The control group mice were gavaged with water, while the mice in the other three groups (the SP, *L. reuteri*, and SP-*L. reuteri* groups) were gavaged with the SP suspension, the *L. reuteri* suspension, or the SP-*L. reuteri* suspension. After 14 days of administration, the animals were evaluated and sacrificed. Blood was taken from the retroorbital plexus, and major organ (heart, lung, liver, spleen, and kidney) tissues were collected. The organs were weighed, embedded in paraffin, sectioned, and stained with HE. The organ indices were calculated using the following equation: organ index = organ weight/body weight.

### Cytokine detection

The levels of inflammatory mediators (IL-6, LPS and TNF-α) and oxidative stress indicators (MPO, MDA) in the serum and intestinal tissue were measured using indicated an ELISA kit according to the manufacturer's instructions (Wuhan Service Technology Co., Ltd., China).

### Determination of the intestinal colonization ability of SP-*L. reuteri*

SP-*L. reuteri* was prepared after labelling *L. reuteri* bacteria with DIR dye. The mice (BTBR, male, 6 weeks old) were randomly divided into two groups (n = 6), after which the abdominal hair was removed. Fluorescence imaging of the mice was performed via an animal fluorescence imaging system at 2, 4, 6, 8, 10, 16, 24, and 32 h after gavage with equal amounts of *L. reuteri* or SP-*L. reuteri*, and gastrointestinal tract tissue was isolated to observe the fluorescence distribution.

The mice (BTBR, male, 6 weeks old) were divided into 2 groups (n = 6) that received intragastric administration of *L. reuteri* or SP-*L. reuteri* (10^8^ CFU, 10 mg/kg/day) for 3 days. Faeces were collected, and the mice were sacrificed. Intestinal tissues such as the jejunum, colon, and ileum were collected, and the contents of the tissues were removed. Faeces and tissues were separately homogenized and diluted with 1 mL of PBS. The homogenate (40 µL) was spread on MRS agar medium overnight and cultured at 37 °C, followed by counting and analysis.

### Treatment of ASD model mice with SP-*L. reuteri*

BTBR mice (4 weeks, male) were randomly divided into 4 groups (n = 10-12): the H_2_O, SP, *L. reuteri,* and SP-*L. reuteri* groups. The mice in the H_2_O group were gavaged with water, while the mice in the other three groups (the SP group, *L. reuteri* group and SP-*L. reuteri* group) were gavaged with the SP suspension, *L. reuteri* suspension, or SP-*L. reuteri* suspension, respectively. Feces sample and blood collection, behavioural assays, and tissue collection were initiated 4 weeks after the beginning of the treatment period.

### Behavioural experiments

All behavioural experiments were conducted on mice aged 8-9 weeks, unless otherwise stated. Mice were habituated to the experimenter for 3 days prior to the start of the behavioural experiment. All of the experiments were conducted during the light cycle.

#### Three-chamber test

Social behaviour in the 3-chamber test was assessed as previously described 2. Briefly, mice were first habituated for 10 min in an empty Plexiglas arena (100 cm × 100 cm × 50 cm) divided into three equally sized interconnected chambers (left, centre, right). After adaptation, empty cups and unfamiliar mice (S1) were placed in the left and right chambers, respectively, so that the subject could interact with the empty wire cup (empty) or the wire cup containing S1 mouse. The control mice were C57BL/6J mice matched in age and sex to the subject mouse. Sociability was evaluated during a second 10-min period, and interaction time was scored by measuring the time that the subject sniffed or climbed onto an empty cup or a cup containing the stranger mouse. Preference for social novelty was assessed by introducing a second stranger mouse (S2) into the previously empty wire cup over a third 10-min period. The time spent interacting with either S1 or S2 was measured as described above. Interaction time was scored using automated AnyMaze software.

#### Self-grooming test

Prior to the self-grooming test, the mice were allowed to acclimate to the test chamber for 60 min. The experiment was conducted in a new cage without bedding. The cumulative time the mice spent grooming themselves was measured over the next 10 min after a 10 min habituation period to the test cage 22.

#### Open field test

The open field test (OFT) was performed according to the methods described in our previous study 23. Before the OFT, the mice were habituated to the testing room for 1 day. The open field consisted of a box (100 cm × 100 cm × 50 cm) containing peripheral and central sectors. Each mouse was placed individually in the central sector, and its locomotion was recorded. Behavioural parameters recorded during the 30-min test period included the total distance travelled and the time spent in the central and peripheral zones. After each trial, the apparatus was cleaned with 75% ethanol.

#### Marble-burying test

The marble-burying test was performed as previously described 24. We used a cage (25 × 40 cm × 18 cm) containing fresh bedding to a depth of 5 cm. Mice were allowed to acclimate for 30 min, after which any faeces and urine were removed. Next, we placed twenty black marbles (25 mm in diameter) in 5 × 4 equidistant rows on the bedding of the same cage, and exploration continued for 30 min. At the end of this 30-min period, the number of marbles buried (2/3 covered by bedding) was counted. Testing was performed under dim light.

### Quantitative PCR

Genomic DNA from intestinal samples were extracted using a Genome Extraction Kit (Beyotime, Shanghai, China) according to the manufacturer's instructions. The extracted DNA was used as a template to amplify with specific primers. A complete list of primer sequences is provided in Table S2.

### 16S rRNA gene sequencing of feces samples

16S rRNA gene sequencing was performed by Shaanxi Weifan Biotechnology Co., Ltd., as previously described 25. Feces genomic DNA was extracted and the V4 region of the 16S rDNA was amplified via PCR using the primers F (CCTAYGGGRBGCASCAG) and R (GGACTACNNGGGTATCTAAT). A next-generation sequencing library was constructed and sequenced using the iSeq platform (Illumina). The sequences obtained by sequencing were subjected to quality filtering, modification and chimaera removal, followed by OTU clustering, annotation and a series of analyses.

### Intestinal barrier integrity

#### Histology analysis

The intestinal tissue was removed, opened longitudinally, rinsed to clean internal contents, and rolled over the full length to obtain a 'Swiss roll', which was then fixed in 4% formaldehyde in PBS for 1 h at 25 °C. Fixed tissues were dehydrated by gradually soaking in alcohol and xylene and then embedded in paraffin. The paraffin-embedded specimens were cut into 5 µm sections, stained with haematoxylin-eosin, and viewed with a digital inverted light microscope (NX50, Thermo Fisher Scientific, China).

#### Western Blot analysis

All samples were isolated by the protein extraction reagent (KeyGen Biotech, Nanjing, China). Anti-ZO-1 (1:1000, Affinity Biosciences, China), anti-claudin1 (1:2000, Abcam, Cambridge, England), β-actin (1:1000, Immunoway, China) and GAPDH (1:4000, Immunoway, China) primary antibodies were incubated with the protein samples overnight. Next, the secondary antibodies (Beyotime, Shanghai, China) was used to incubate the samples for 60 min, and the ECL chemiluminescence detection system (Bio-Rad, Hercules, CA, USA) was performed to detect the protein bands. Image analysis was performed via NIH ImageJ v1.53c software (Bethesda, MD, USA).

#### Immunofluorescence staining

Mice were anaesthetized, 4% paraformaldehyde was perfused intracardially, and brains were harvested. The brains were postfixed with 4% paraformaldehyde for 12 h, dehydrated in 30% sucrose solutions, and embedded in OCT. Coronal sections of the paraventricular thalamic nucleus region were performed using a cryostat microtome (model 1950, Leica) with a thickness of 30 µm. Free-floating sections were washed in PBS, blocked with a buffer containing 5% bull serum albumin and 0.3% Triton X-100 for 1 h, incubated with primary antibodies (anti-c-fos/anti-Iba-1) at 4 °C overnight, washed three times in PBS, and then incubated with a secondary antibody solution for 1 h at 37 °C. Secondary antibodies (goat anti-rabbit, Alexa Fluor 488, 1:500) were applied for 2 h at room temperature. Sections were then washed three times and mounted on microscope slides. Images were captured under fluorescence microscopy (NX50, Thermo Fisher Scientific, China).

### Statistical analysis

Spearman's rank correlation coefficient was used for linear correlation analysis. The statistical analysis was performed using GraphPad Prism 9. The statistical significance was determined using Student's t test (two-tailed) or two-way ANOVA followed by Tukey's multiple comparisons. Differences between the experimental groups and control groups were considered statistically significant at *p* < 0.05.

## Results

### Construction and characterization of SP-bacteria

**Figure 1:**
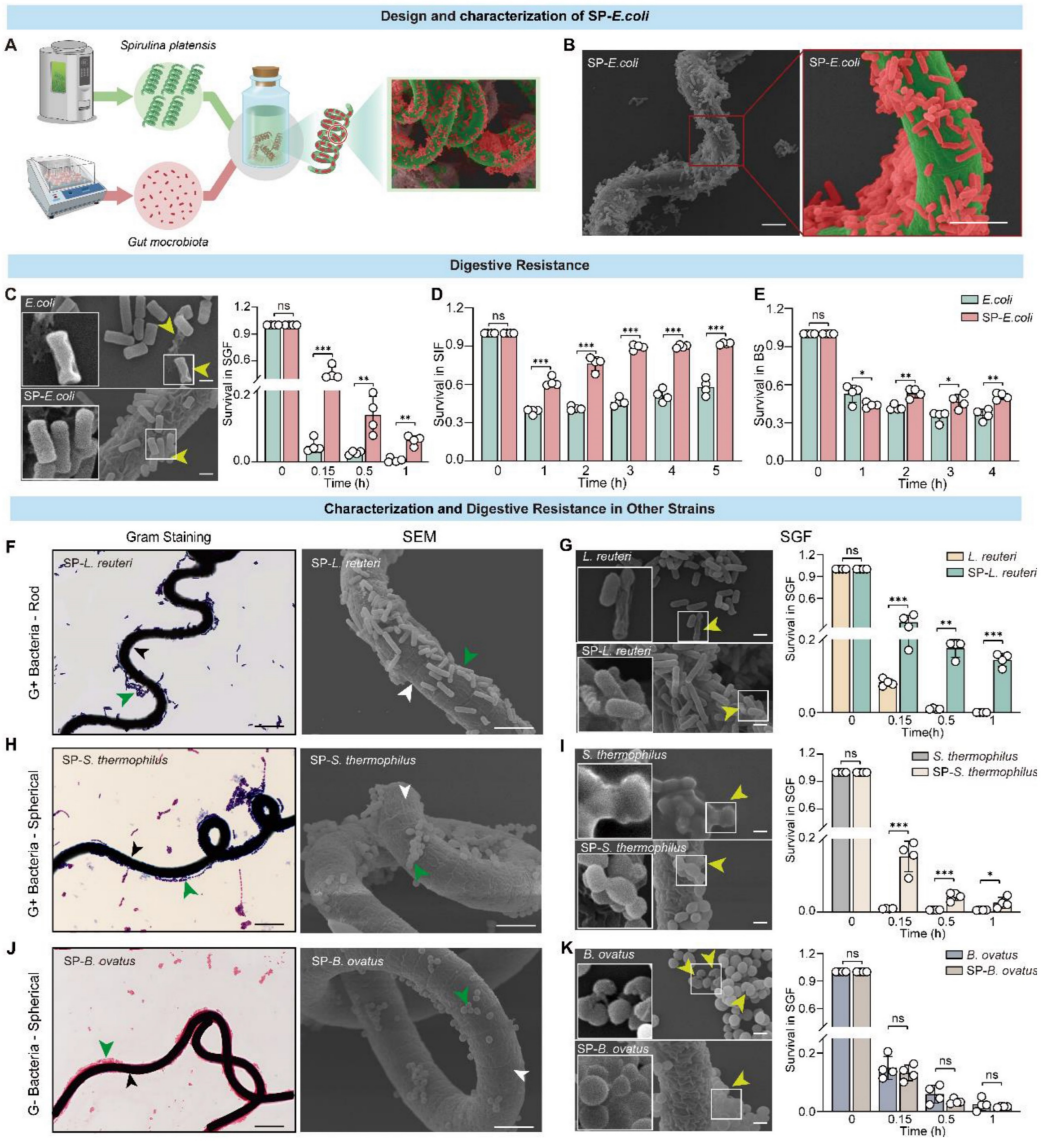
***E. coli* and multiple additional types of bacteria can attach to the surface of SP and tolerate the gastrointestinal environment *in vitro*.** A, Schematic illustration of SP-*E. coli* constructed by coincubating SP and *E. coli* under specific conditions. B, Representative SEM image of SP-*E. coli*. C-E, (C) SEM image and bacterial survival rate of SP-*E. coli* and* E. coli* after exposure to SGF (pH 1.2, supplemented with pepsin). Bacterial survival rate of SP-*E. coli* and* E. coli* after exposure to (D) SIF (pH 6.8, supplemented with trypsin) or (E) bile salts (0.3 mg/mL) for a specific period. F-K, Typical Gram-stained (left) and SEM (right) images of (F) SP-*L. reuteri*, (H) SP-*S. thermophilus*, and (J) SP-*B. ovatus*. The white and black arrows indicate SP, green arrows indicate bacteria. SEM images (left) and bacterial survival rates (right) of (G) SP-*L. reuteri*, (I) SP-*S. thermophilus*, and (K) SP-*B. ovatus* after exposure to SGF. Yellow arrows indicate damaged bacteria. *L. reuteri*, *S. thermophilus*, and* B. ovatus* were used as controls. Scale bar = 5 µm. The error bars represent the standard deviations (n = 4). Statistical significance was assessed using two-way ANOVA (ns: not significant, * *p* < 0.05, ** *p* < 0.01, *** *p* < 0.001).

To provide a proof of principle, we employed SP as the natural living microcarrier, and the commonly used model strain *Escherichia coli* carrying GFP (*E. coli*) was selected for use in constructing an oral delivery system. The SP surface was loaded with *E. coli* to produce SP-*E. coli* (Figure 1A). The optimized culture conditions are shown in Table 1. Natural SP exhibits a green colour and a standard spiral shape and emits strong red fluorescence when exposed to light at an appropriate wavelength for excitation (~605 nm) (Figure S1A) 16. Gram staining of SP-*E. coli* showed that many *E. coli* were attached to the surface of the SP (Figure S1B). Fluorescence microscopy revealed that SP-*E. coli* emitted both red fluorescence (from SP, chlorophyll) and green fluorescence (from *E. coli*) around the red surface (Figure S1C). Moreover, the viability of SP-*E. coli* was not affected, and the growth curve of SP-*E. coli* was not different from that of *E. coli* (Figure S2A-B). Scanning electron microscopy (SEM) confirmed that *E. coli* was efficiently loaded onto the SP surface (Figure 1B).

The first challenge after oral administration is safety in the gastrointestinal environment. SP-*E. coli* was coincubated with simulated gastric fluid (SGF, pH 1.2), simulated intestinal fluid (SIF, pH 7), and bile salts (BS, 0.3 mg/mL). The survival rate of the bacteria after incubation was assessed by counting the number of bacteria at different time points. The survival rate of SP-*E. coli* was significantly greater than that of *E. coli* at the specified time points (Figure 1C-E). SEM imaging revealed that after coincubation with SGF for 10 min, a small number of bacteria in the SP-*E. coli* group exhibited membrane shrinkage and depression, while some bacteria in the *E. coli* group presented disrupted membranes and content leakage (Figure 1C). The presence of SP can also protect the *E. coli* structural integrity in the SIF and BS (Figure S1D-E). The alkaline substances released from SP, which is rich in alkaline minerals such as Na, K, Ca, and Mg and can neutralize gastric acid 26. Due to the lower amount of SP in the SP-intestinal bacteria, it cannot significantly change the pH value of SGF (Figure S1F-G). Therefore, the protection mechanism of SP against bacteria in gastric acid may primarily be the reduction in the contact area of bacteria with SGF after adhering to SP. These results indicate that SP-*E. coli* can improve the survival rate of bacteria in the gastrointestinal environment.

Additionally, bacterial vitality is also an important factor for intestinal colonization. Bacterial viability was determined by constructing a growth curve after coincubation with the simulated gastrointestinal fluid. As shown in Figure S3A, in contrast with *E. coli*, which lost its proliferative ability after coincubation with SGF for 30 min, SP-*E. coli* still exhibited proliferative activity. Moreover, there was no significant difference in the growth capacity of SP-*E. coli* or *E. coli* alone after incubation with SIF or BS for 1 h (Figure S3B-C). Overall, SP-*E. coli* improved bacterial viability in SGF.

We further investigated the generalizability of the results to other bacteria loaded onto the SP surface. Several bacterial strains with different morphologies, including gram-positive rod-shaped *Lactobacillus reuteri* (*L. reuteri*) and spherical *Streptococcus thermophilus* (*S. thermophilus*), as well as gram-negative spherical *Bacteroides ovatus* (*B. ovatus*), were tested for their ability to attach to SP. Similarly, coincubation of bacteria with SP did not affect bacterial viability or proliferation (Figure S2C-H). Gram staining and SEM imaging confirmed that bacteria with different shapes could attach to the surface of the SP to generate SP-bacteria (Figure 1F, H, J). The tolerance of these strains was also tested, and SP-*L. reuteri* and SP-*S. thermophilus* exhibited significantly improved survival rates in SGF (Figure 1G, I), SIF (Figure S2I, J), and BS (Figure S2L, M). The survival rates of *B. ovatus* and SP-*B. ovatus* were similar in SGF and BS (Figure 1K and Figure S2K, N). The bacterial morphology of SP-*L. reuteri*, SP-*S. thermophilus*, and SP-*B. ovatus* under SGF exposure was more intact than that of the unmodified bacteria (Figure 1G, I, K). Similarly, SP-*L. reuteri*, SP-*S. thermophilus*, and SP-*B. ovatus* exhibited improved bacterial viability in SGF but not in SIF or BS (Figure S3D-L).

Notably, the SEM results showed that rod-shaped bacteria (*L. reuteri* and *E. coli*) had greater adhesion efficiency on the surface of SP than did spherical bacteria (*S. thermophilus* and *B. ovatus*). We speculated that this difference may be due to the difference in contact surface area. Therefore, the microalga GY-D16 *Nostoc* sp., which has a smaller diameter and similar length to SP, was selected as a carrier for spherical bacteria (*B. ovatus*). SEM demonstrated that *B. ovatus* could efficiently adhere to the surface of *Nostoc* sp. (Figure S4)*,* further confirming the versatility of microalgae-assisted intestinal bacteria delivery systems.

### Enhanced intestinal adhesion of SP-*E. coli*

**Figure 2:**
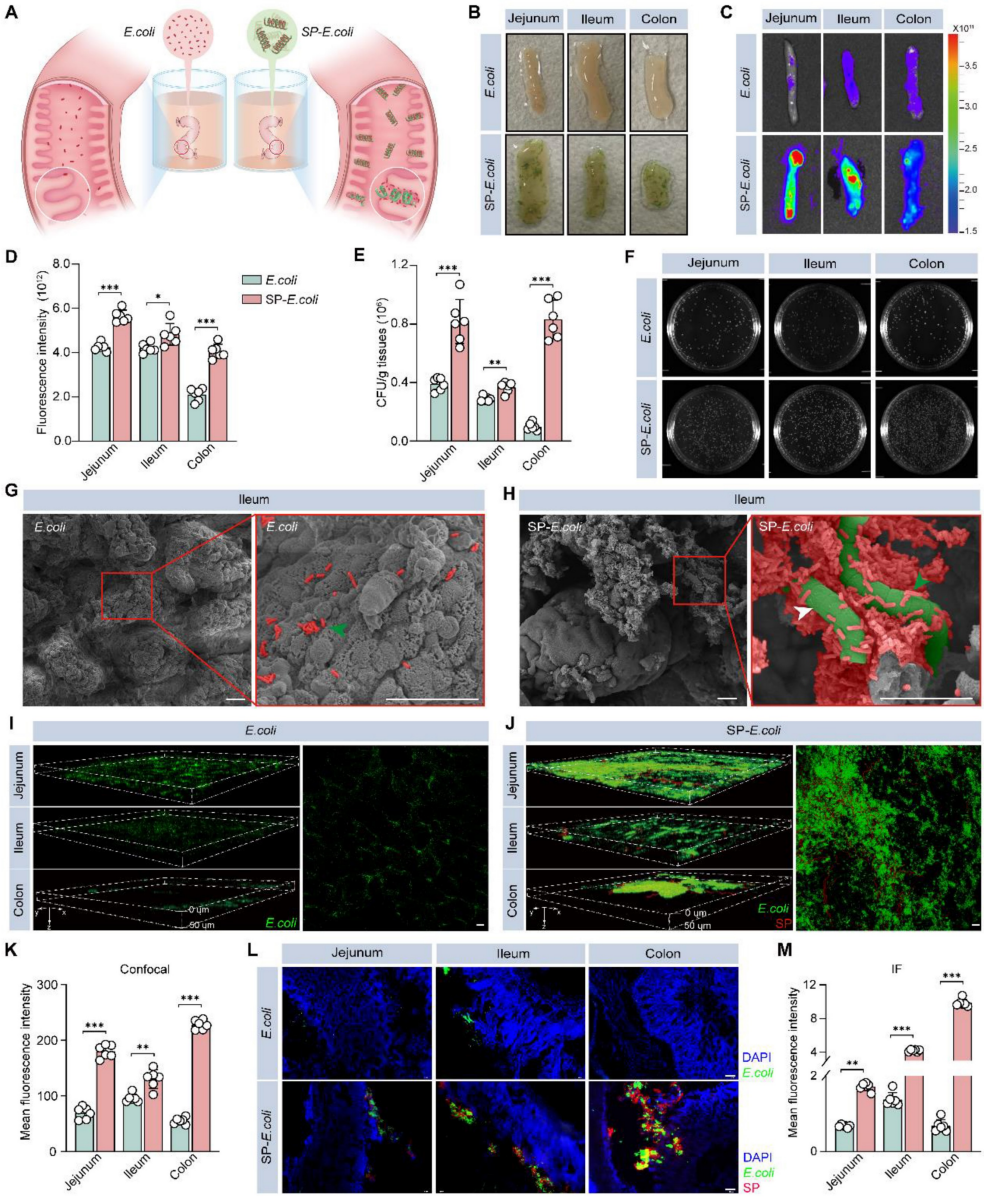
** SP-*E. coli* exhibits greater engraftment efficiency in the gut lumen than did *E. coli in vitro*.** A, Schematic illustration of bacterial adhesion in isolated intestinal tissue. The mouse intestine was removed and separated into intestinal segments with a length of 1.5 cm, after which the contents were removed. An equal amount of 150 µL of *E. coli* or SP-*E. coli* was injected into the intestinal lumen, and the intestinal tissue was ligated at both ends. After incubation for 30 min, the samples were rinsed to completely remove unattached bacteria. B-F, (B) Digital images, (C) fluorescence imaging images and (D) corresponding statistical analysis of fluorescence intensity, (E) statistical analysis and (F) LB agar plates for colony counting of intestinal segments (jejunum, ileum, colon) incubated with *E. coli* or SP-*E. coli*. G-H, SEM image of the ileum villi exposed to (G) *E. coli* or (H) SP-*E. coli*. Scale bar = 20 µm. The green and white arrows indicate* E. coli* and SP, respectively. I-K, Representative scanning confocal microscopy images of intestinal segments exposed to (I) *E. coli* and (J) SP-*E. coli* and (K) statistical analysis of fluorescence intensity. Scale bar = 50 µm. L-M, (L) Fluorescence imaging images and (M) corresponding statistical analysis of fluorescence intensity. The error bars represent the standard deviations (n = 6). Statistical significance was assessed using two-way ANOVA (ns: not significant, * *p* < 0.05, ** *p* < 0.01, *** *p* < 0.001).

The long helical structure and high surface area of SP make it more readily captured by intestinal villi and maintain close contact with the intestinal tissue 14, 27, and the charge and adsorption properties of SP may allow it to adsorb on the surface of intestinal tissue. To study the adhesion of SP-*E. coli*, we sampled and dissected the intestines of the mice and exposed them to SP-*E. coli* or *E. coli* suspensions (Figure 2A). As shown in Figure 2B, a large amount of SP-*E. coli* adhered to the surface of the intestine. The fluorescence signals of endogenous GFP expressed by *E. coli* were recorded using live animal imaging. The gut (jejunum, ileum, and colon) after exposure to SP-*E. coli* showed a stronger fluorescence signal than that after exposure to *E. coli* (Figure 2C), with signal intensities 1.3, 1.1, and 2.0 times greater, respectively, than those after exposure to *E. coli* (Figure 2D). Intestinal segments were further homogenized for plate-based counting. The numbers of bacteria adhering to the jejunum, ileum, and colon after SP-*E. coli* exposure were more than 2.0, 1.3, and 8.4 times greater than those after *E. coli* exposure, respectively (Figure 2E). A representative image of an LB agar plate used for colony counting is shown in Figure 2F. Notably, similar results were obtained with gastric tissues and duodenum. Gastric tissues and duodenum exposed to SP-*E. coli* had a greater fluorescent signal and a greater number of bacteria (Figure S5A-E). Since the jejunum, ileum, and colon are the primary sites for microbial colonization and nutritional absorption by the host, they are generally considered key regions for studying the colonization effects on intestinal tissues 28-30. The adhesion efficiency of SP-*E. coli* in the stomach and duodenum is relatively low compared with that in other intestinal tissues (jejunum, ileum, and colon). So, we focus primarily on the small intestine and colon tissues in the follow experiments.

Next, we visualized the adhesion of SP-*E. coli* to the intestine. SEM images of the ileum tissues showed that SP-*E. coli* adhered to intestinal villi more efficiently than did *E. coli* (Figure 2G-H). The bacteria adhering to the intestines are exogenous, not original (Figure S6A). The intestinal tract was scanned using laser scanning confocal microscopy (LSCM) to observe GFP signals. The fluorescence signals were scattered in the intestinal villi at a depth of 50 µm (Figure 2I-J), and the signal intensity and density distribution of intestinal tissue in the SP-*E. coli* group were greater and broader, respectively, than those in the *E. coli* group (Figure 2K). In addition, frozen intestinal tissue sections were examined via fluorescence microscopy. SP-*E. coli* with both red and green fluorescence was attached to the surface of the intestinal villi (Figure 2L), and the fluorescence signal of the intestine exposed to SP-*E. coli* was significantly greater than that of the intestine exposed to *E. coli* (Figure 2M). Taken together, these data suggested that SP-*E. coli* can significantly improve adhesion efficiency in the intestine.

In addition, we also detected the intestinal adhesion properties of SP-*B. ovatus* and SP-*S. thermophilus in vitro* by SEM and bacterial count assays. Compared with those of *S. thermophilus* and* B. ovatus*, the numbers of SP-*B. ovatus* and SP-*S. thermophilus* attached to the intestinal surface were significantly greater (Figure S6B-G). These results confirmed the universality of intestinal bacteria-loaded SP in enhancing intestinal adhesion *in vitro*.

### Increased intestinal colonization of SP-*E. coli*

**Figure 3:**
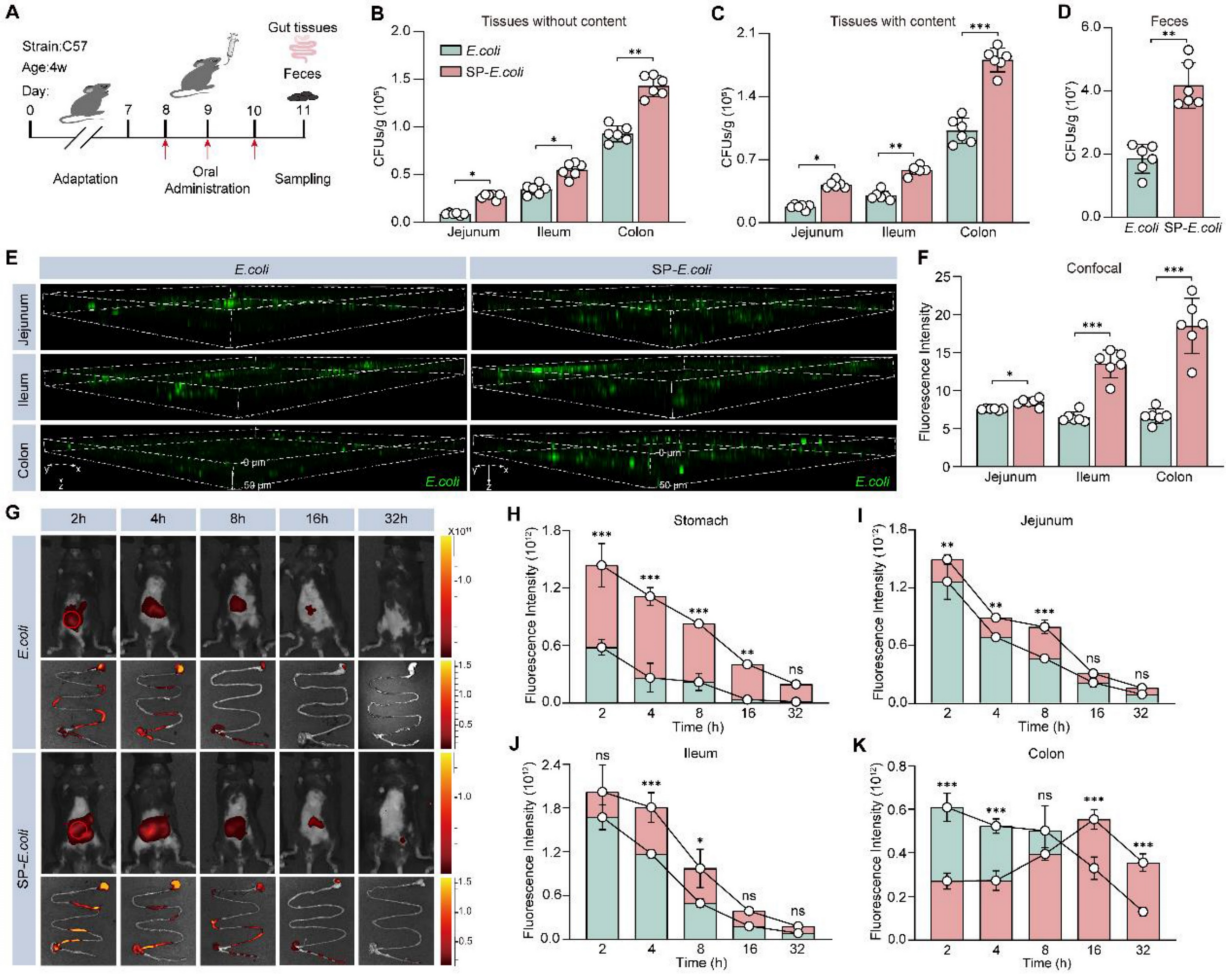
** SP-*E. coli* increases intestinal colonization and prolongs residence time in the intestinal tract in mice.** A, Schematic diagram of the experimental design. B-D, Bacterial counts in the intestinal tract without (B) or with (C) contents and (D) feces collected 96 h after oral gavage of 3 × 10^8^ CFU of *E. coli* or SP-*E. coli*. E-F, (E) Scanning confocal microscopy images and (F) statistical analysis of the fluorescence intensity of intestinal tissue. Scale bar = 100 µm. G-K, (G) Representative live animal fluorescence images and fluorescence intensities of (H-K) intestines sampled at different time periods after oral gavage of 3 × 10^8^ CFU of *E. coli* or SP-*E. coli*. The error bars represent the standard deviations (n = 6). Statistical significance was assessed using two-way ANOVA or Student's two-tailed t-test (ns: not significant, * *p* < 0.05, ** *p* < 0.01, *** *p* < 0.001).

In a preliminary experiment, 3 days after oral administration of the bacteria, the intestinal colonization efficiency of the exogenous bacteria plateaued (Figure S7A). To quantify colonization efficiency, the mice were sacrificed on day 3, and intestinal tissue and feces were collected for further colony counting analysis (Figure 3A). The colony numbers in the jejunum, ileum, and colon without or with contents with SP-*E. coli* treatment were significantly greater than those with *E. coli* treatment (Figure 3B-C). There were 2.2-fold more colonies with SP-*E. coli* treatment in the feces samples than with *E. coli* treatment (Figure 3D). LSCM images of intestinal tissue showed that the fluorescence signal in the SP-*E. coli* treatment group was stronger and more widely distributed than that in the *E. coli* treatment group (Figure 3E). The fluorescence intensities in the jejunum, ileum, and colon after SP-*E. coli* treatment were 1.1, 2.1, and 2.7 times greater, respectively, than those after *E. coli* treatment (Figure 3F). The intestinal tissue was further subjected to cryo-sectioning for fluorescence microscopy. The intestinal tissue of the SP-*E. coli* treatment group had a more intense green fluorescence signal (Figure S7B). In summary, these results supported the conclusion that SP-*E. coli* has high intestinal colonization efficiency in mice.

To further evaluate the colonization efficiency of SP-*E. coli* in the intestine and visualize its distribution throughout the whole intestinal tract, *E. coli* were labelled with DIR dye, and then SP-*E. coli* labelled with DIR were prepared. Live animal fluorescence imaging after administration revealed that the fluorescence intensity in the abdominal area of mice treated with SP-*E. coli* was significantly greater than that in the abdominal area of mice treated with *E. coli* at 4 h after gavage (Figure 3G, Figure S7C). The gastrointestinal tissues of the mice were isolated and subjected to fluorescence imaging. The fluorescence signals of the stomach, duodenum, jejunum, cecum, and ileum tissues were stronger in the SP-*E. coli* treatment group than in the *E. coli* treatment group (Figure 3H-J, Figure S7D-E). The fluorescence signals of the colon in the SP-*E. coli* treatment group first increased but then decreased (Figure 3K). We speculated that this was due to the slow transport of SP-*E. coli* in the gastrointestinal tract of mice. These data demonstrated that SP-*E. coli* had a lower transit rate and greater colonization efficiency and retention ability in the gastrointestinal tract of mice than did *E. coli*.

### Enhanced intestinal adhesion and colonization of SP-*L. reuteri*

**Figure 4:**
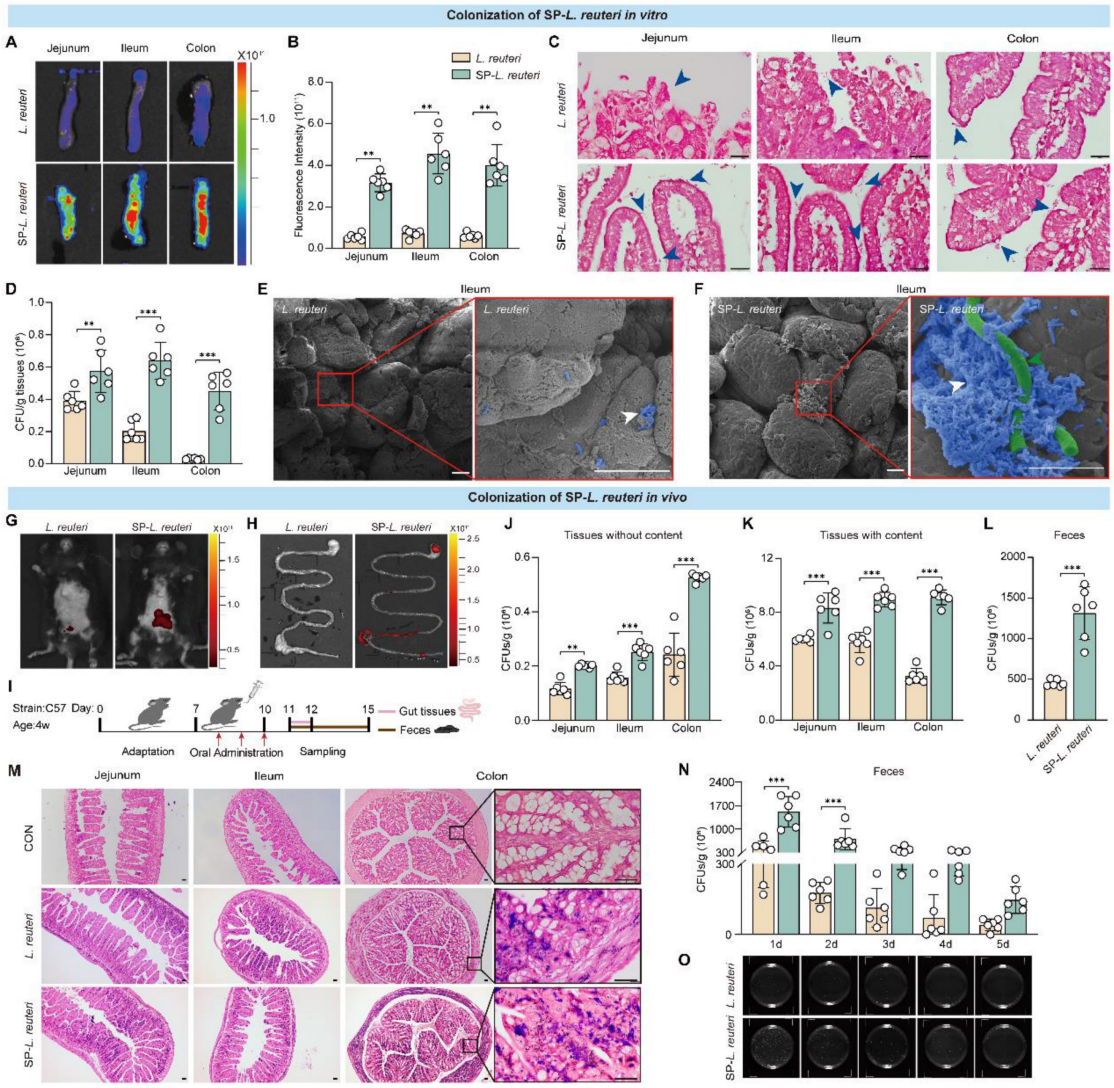
** SP-*L. reuteri* exhibits stronger intestinal adhesion and colonization both *in vivo* and *in vitro*.** A-D, (A) Representative fluorescence images, (B) fluorescence intensities, (C) Gram staining, and (D) bacterial counts of the jejunum, ileum, and colon tissues after culture with equivalent amounts of native *L. reuteri* or SP-*L. reuteri* for 30 min. The blue arrows indicate bacteria. E-F, SEM images of the ileum villi exposed to (E) *L. reuteri* or (F) SP-*L. reuteri*. The white arrows indicate bacteria adhering to intestinal tissue, green arrow indicate SP. Scale bar = 20 µm. G-H, Fluorescence images of (G) mice and (H) the gastrointestinal tract 24 h after oral administration. I, Flow chart of the *in vivo* experimental design. J-L, Bacterial counts of the intestine (J) without or (K) with contents and (L) feces samples collected 96 h after oral gavage of 3 × 10^8^ CFU of *L. reuteri* or SP-*L. reuteri*. M, Gram staining of intestinal tissue. Scale bar = 20 µm. N-O, (N) Bacterial count and (O) image of LB agar plates used for determining the colony counts of feces samples collected 5 consecutive days after oral gavage of 3 × 10^8^ CFU of *L. reuteri* or SP-*L. reuteri* for 3 days. The error bars represent the standard deviations (n = 6). Statistical significance was assessed using two-way ANOVA or Student's two-tailed t-test (ns: not significant, ** *p* < 0.01, *** *p* < 0.001).

Previous studies have shown that *L. reuteri* supplementation can effectively alleviate the core symptoms of ASD in mice 5. We next tested the colonization ability of SP-*L. reuteri*. *L. reuteri* was labelled with DIR dye, after which SP-*L. reuteri* labelled with DIR was prepared. C57BL/6J mouse intestinal tissues were prepared and exposed to SP-*L. reuteri* or *L. reuteri* suspensions. After incubation for 30 min, the intestinal tissue was washed three times with PBS to remove nonattached bacteria. Tissue fluorescence imaging and statistical analysis were performed. Compared to that of *L. reuteri*, the fluorescence signal of intestinal tissue after SP-*L. reuteri* exposure was stronger (Figure 4A-B). Gram staining of intestinal tissue revealed more *L. reuteri* adhering to the intestinal villi after SP-*L. reuteri* treatment than after *L. reuteri* treatment (Figure 4C). After 30 min of exposure to different environments, the number of bacteria adhering to the intestinal tract after SP-*L. reuteri* exposure was significantly greater than that after *L. reuteri* exposure (Figure 4D). The adhesion of bacteria to the ileum villi was visualized through SEM. The ileum villi of the *L. reuteri* treatment group exhibited only a small amount of bacterial adhesion, whereas many bacteria were adhered to the intestinal villi of the SP-*L. reuteri* treatment group (Figure 4E-F). These results were consistent with those obtained for SP-*E. coli*, verifying that SP-*L. reuteri* also efficiently adhered to intestinal tissue.

Next, we tested the colonization of mice by SP-*L. reuteri in vivo*. The fluorescence signal of DIR in the SP-*L. reuteri* treatment group was greater than that in the *L. reuteri* treatment group at 24 h after intragastric administration (Figure 4G-H). To further determine whether SP-*L. reuteri* can enhance intestinal colonization *in vivo*, the mice were sacrificed 3 days after oral administration, and jejunum, ileum, colon, and feces samples were collected for bacterial quantification (Figure 4I). Jejunum, ileum, and colon tissues without their contents were homogenized for bacterial enumeration. The number of bacteria adhering to the jejunum, ileum, and colon in the SP-*L. reuteri* group was 1.7, 1.6, and 2.2 times greater than that in the *L. reuteri* group, respectively (Figure 4J). The colonization of the intestine by SP-*L. reuteri* was also measured by bacterial enumeration of intestinal tissue containing contents and feces. The number of bacteria colonizing the gut was significantly greater in the SP-*L. reuteri* group than in the *L. reuteri* group (Figure 4K-L). In addition, Gram staining of intestinal tissue further confirmed the high intestinal colonization ability of SP-*L. reuteri* (Figure 4M). To investigate the survival and retention of SP-*L. reuteri*, feces were collected for 5 consecutive days to monitor bacterial colonization. The results showed that SP-*L. reuteri* had a longer retention time than *L. reuteri* and persisted for up to 5 days (Figure 4N). A representative LB agar plate is shown in Figure 4O.

Moreover, we examined the biosafety of SP-*L. reuteri* in C57BL/6J mice. After 14 days of gavage treatment, there was no significant difference in the organ indices (Figure S8A) or the expression of cytokines reflecting inflammation and oxidative stress in the serum (Figure S8B-D) among the mice in each group. The HE staining results also showed that there was no damage to the tissue structure (Figure S8E).

### Treatment with SP-*L. reuteri* alleviated core symptoms in ASD model mice

**Figure 5:**
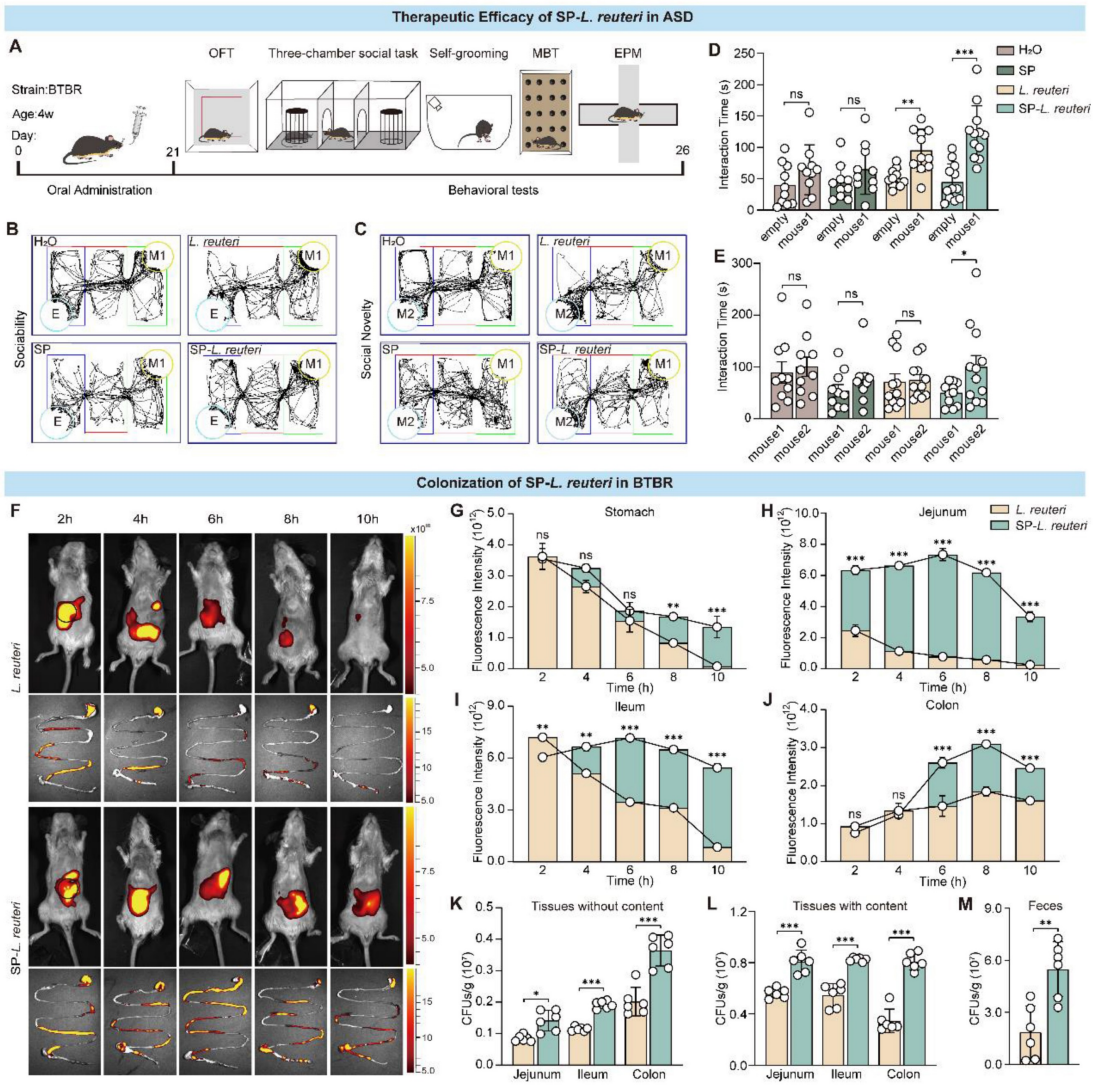
** SP-*L. reuteri* ameliorates social deficits in BTBR mice and prolongs the intestinal residence time of *L. reuteri*.** A, Experimental flow chart of the treatment of ASD model mice with SP-*L. reuteri*. B-E, the social behaviour of BTBR mice was assessed via the 3-chamber test. (B, C) Representative animal tracks and (D, E) interaction time of sociability and social novelty. The error bars represent the standard deviations (n = 10-12). F-J, (F) Representative live animal fluorescence images and fluorescence intensities of the intestine (G, stomach; H, jejunum; I, ileum; J, colon) sampled at different time periods after oral gavage of 3 × 10^8^ CFU of *L. reuteri* or SP-*L. reuteri*. K-M, Bacterial counts in the intestinal tract (K) without and (L) with contents and (M) feces collected 96 h after oral gavage of 3×10^8^ CFU of *L. reuteri* or SP-*L. reuteri* in BTBR mice. The error bars represent the standard deviations (n = 6). Statistical significance was assessed using two-way ANOVA or Student's two-tailed t-test (ns: not significant, * *p* < 0.05, ** *p* <0.01, *** *p* < 0.001).

Having confirmed the robust intestinal adhesive properties of SP-*L. reuteri*, we next investigated whether this formulation could serve as an enhanced therapy for mental disorders. To explore whether SP-*L. reuteri* had therapeutic efficacy against ASD, 4-week-old BTBR mice were treated with SP-*L. reuteri* or *L. reuteri* for 4 weeks, and behavioural tests were subsequently performed (Figure 5A). In a three-chamber test of sociability, compared with those in the H_2_O and SP groups, the mice in the SP-*L. reuteri* and *L. reuteri* groups spent more time in the area where the first stranger mouse (M1) was located than in the empty cage (Figure 5B, D). During the social novelty stage, mice in the SP-*L. reuteri* group spent a significantly longer time sniffing new mice (M2) than familiar mice (M1) (Figure 5C, E). These results indicated that SP-*L. reuteri* can ameliorate sociability and social novelty. Compared with *L. reuteri*, SP-*L. reuteri* had a more prominent therapeutic effect on ASD model mice.

There was no significant difference in the feces water content (Figure S9A), which reflects their safety. It was found that SP-*L. reuteri* treatment did not affect the repetitive stereotyped and anxiety behaviors of BTBR mice using a self-grooming, marble-burying test, open field test, and elevated test (Figure S9B-I). Furthermore, we compared the therapeutic effects of the simple complex of SP and *L. reuteri* (SP+*L. reuteri*) and SP-*L. reuteri*. The results indicated that the SP+*L. reuteri* treatment improved sociability in BTBR mice but did not impact their social novelty (Figure S9J-K), which is similar to the results from the *L. reuteri* group. SP-*L. reuteri* notably enhanced both the sociability and social novelty of BTBR mice (Figure S9J-K). These findings rule out the role of the simple combination of SP and *L. reuteri*, further supporting the therapeutic effect of SP- *L. reuteri*. Therefore, compared with *L. reuteri*, SP-*L. reuteri* significantly ameliorated the core symptoms of ASD.

We evaluated th*e L. reuteri* DNA expression using qPCR analysis. The results revealed that the expression of intestinal *L. reuteri* in the SP-*L. reuteri* treated group was significantly higher than that in the other groups (Fig S9L, M). The correlations between the level of intestinal* L. reuteri* and social behaviour were further analysed. The results showed that the level of ileum* L. reuteri* was significantly positively correlated with both sociability and social novelty, and the level of colonic* L. reuteri* was significantly positively correlated with social novelty after *L. reuteri* and SP-*L. reuteri* treatments in BTBR mice (Figure S9N-Q). This result proves that* L. reuteri* colonization ability is positively related to social behaviour.

Intestinal morphological data from ASD model mice revealed that their intestinal villi are different from those of normal mice 31, 32. The length of the villi and epithelial surface determines the interaction between the intestinal mucosa and luminal contents. We further investigated the colonization of the intestine by SP-*L. reuteri* in BTBR mice. DIR dyes were used to label *L. reuteri*. We verified that SP-*L. reuteri* had a strong ability to adhere to the intestinal tissue of BTBR mice *in vitro* (Figure S9R-S).

Next, after the administration of equal amounts of *L. reuteri* and SP-*L. reuteri* to the BTBR mice, fluorescence signals were recorded at different time points by a small animal imaging system. The fluorescence signal of the SP-*L. reuteri* group was stronger than that of the *L. reuteri* group, and the retention time was longer (Figure 5F, Figure S9T). The fluorescence signals in the stomach and small intestine in the *L. reuteri* group continued to decrease, while the fluorescence signal in the small intestine in the SP-*L. reuteri* group first increased but then decreased (Figure 5G-I). The mean signal intensity in the stomach and intestines was significantly greater in the SP-*L. reuteri* group than in the *L. reuteri* group (Figure 5G-J). Further monitoring of fluorescence intensity in the colon revealed that the fluorescent signal from SP-*L. reuteri* group could last for up to 32 h, with significantly greater fluorescence intensity than that of the *L. reuteri* group (Figure S9U-V). The above results indicated that SP-*L. reuteri* colonization in the intestine was enhanced. Feces and intestinal tissues were collected 3 days after the oral administration of *L. reuteri* or SP-*L. reuteri* for plate counting. As shown in Figure 5K-M, the number of bacteria colonizing the intestinal tissues in the SP-*L. reuteri* group was significantly greater than that in the *L. reuteri* group. The number of bacteria in the feces of the SP-*L. reuteri* group was approximately 3.5 times greater than that in the feces of the *L. reuteri* group. These data suggested that SP-*L. reuteri* has a strong ability to colonize the intestinal of ASD model mice and ameliorate the core symptoms of ASD.

### Supplementation with SP-*L. reuteri* reorganized the gut microbiota in ASD model mice

**Figure 6:**
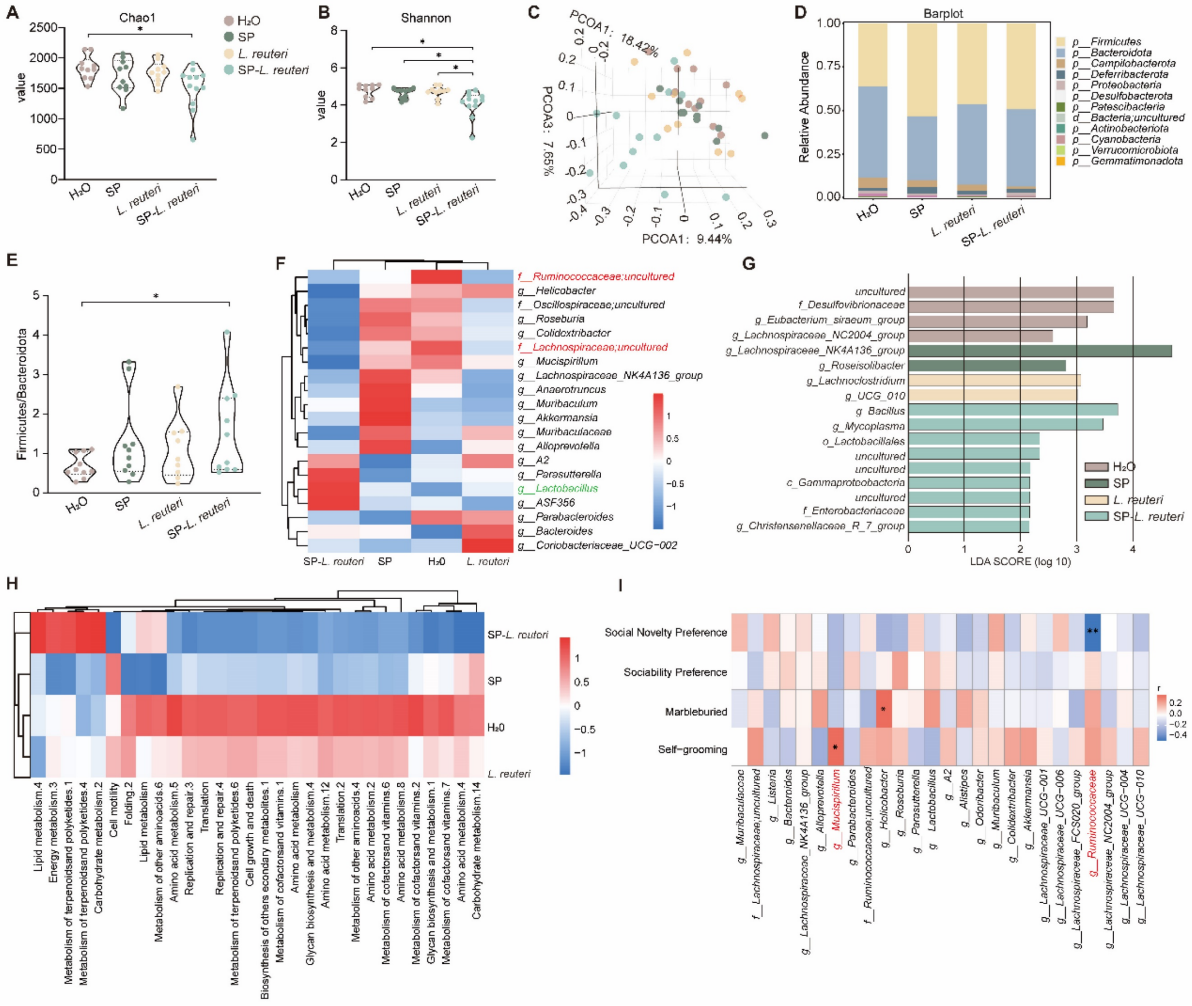
** Supplementation with SP-*L. reuteri* regulates the intestinal microbiota in ASD model mice.** A-B, α-Diversity of the gut microbiota of mice. (A) Chao1 indices reflect the richness of each sample sequence and the (B) Shannon indices reflect the diversity of the intestinal microbial composition. C, Bray‒Curtis-based PCoA analysis of the mouse microbiota community structure. D, Analysis of the relative abundance of the microbiota community at the phylum level. The data are presented as the mean values for ten samples for each group. E, The Firmicutes/Bacteroidetes ratio (F/B ratio) was calculated. F, Clustering heatmap of the relative abundance of microbes at the genus level. The red and blue shading indicate high and low abundance, respectively. Compared with those in the H_2_O group, SP group and* L. reuteri* group, the red font represents genera with increased abundance in the SP-*L. reuteri* group, and the green font represents genera with decreased abundance. G, Linear discriminant analysis effect size (LEfSe) of the feces microbiota of each group. H, Heatmap of the functional prediction analysis of the gut microbiota based on the KEGG database using PICRUSt. The red and blue shading indicate high and low abundance, respectively. I, Heatmap of the Spearman correlation between the top 20 gut microbiota constituents and behaviour. The red and blue dots represent positive and negative correlations, respectively. According to comprehensive data analysis, red font represents bacteria with significant differences in relative abundance and significant correlations with behavioural phenotypes. The error bars represent the standard deviations (n = 10-12 per group). Statistical significance was assessed using one-way ANOVA (ns: not significant, * *p* < 0.05, ** *p* < 0.01).

Alterations in the gut microbiota play a prominent role in behavioural regulation in ASD patients. Compared with C57BL/6J mice, BTBR mice presented social deficit phenotypes (Figure S10A-B). A 16S rRNA gene sequencing analysis revealed that BTBR mice presented notably greater α-diversity than C57BL/6J mice did (Figure S10C-D). PCA revealed that the composition of the intestinal microbiota of BTBR mice significantly differed from that of C57 mice (Figure S10E). Next, we identified the gut bacterial signatures of mice in different groups using 16S rRNA sequencing of the gut microbiota. The microbial diversity and community richness in the SP-*L. reuteri* group were significantly lower than those in the other groups (Figure 6A-B). Principal coordinate analysis (PCoA) was performed to determine the microbiome distance between the different groups, and the results revealed distinct differential clustering of microbial communities between the SP-*L. reuteri* group and the other groups (Figure 6C). Specifically, we noted a reduction in Campylobacteria and an increase in Firmicutes and Deferribacteria abundances in the SP-*L. reuteri* group (Figure 6D). The Firmicutes/Bacteroidetes ratio (F/B ratio) was calculated as a biomarker of gut dysbiosis. The F/B ratio was greater in the SP-*L. reuteri* group than in the H_2_O group (Figure 6E). Further analysis of the relative abundance of gut microbes at the genus level via a heatmap showed that, compared with those in the H_2_O group and *L. reuteri* group, the relative abundances of *Ruminococcaceae*, *Lachnospiraceae*, and *Mucispirillum* in the SP-*L. reuteri* group decreased, while the relative abundances of *Lactobacillus* in the SP-*L. reuteri* group increased (Figure 6F). We then performed linear discriminant analysis (LDA) effect size (LEfSe) analysis to identify the genomic signatures of the gut microbes.* Desulfovibrio* were significantly enriched in the ASD group, while *Bacillus* and* Lactobacillus* were significantly enriched in the SP-*L. reuteri* group (Figure 6G). Combined with the reduction in microbial diversity, these findings led us to speculate that the ameliorative effect of SP-*L. reuteri* on ASD model mice was partly due to the removal of harmful species.

Importantly, differences in microbial community structure also reflect variations in predicted bacterial functions. Prediction analysis of microbial functions revealed that the SP-*L. reuteri* group was enriched mainly in lipid metabolism, energy metabolism, terpenoid and polyketide metabolism, and carbohydrate metabolism. In addition to the above metabolic pathways, amino acid metabolism, translation, replication, and repair were also enriched in the ASD model mice (Figure 6H). We tested for associations (Spearman correlation) between the gut microbiota and the related behavioural indices. The abundance* of Ruminococcaceae* was negatively correlated with social novelty and positively correlated with immune factors (Figure 6I, Figure S10F).* Mucispirillum* abundance was positively associated with grooming behaviour (Figure 6I).

In summary, microbiota analysis revealed that the BTBR mouse microbiota was altered, as characterized by an enrichment of intestinal pathogens and increased bacterial diversity. The addition of SP-*L. reuteri* reduced the abundance of pathogenic bacteria and increased the relative abundance of beneficial bacteria, thereby alleviating social deficits.

### Protective effects of SP-*L. reuteri* on the intestinal barrier and PVT regions

**Figure 7:**
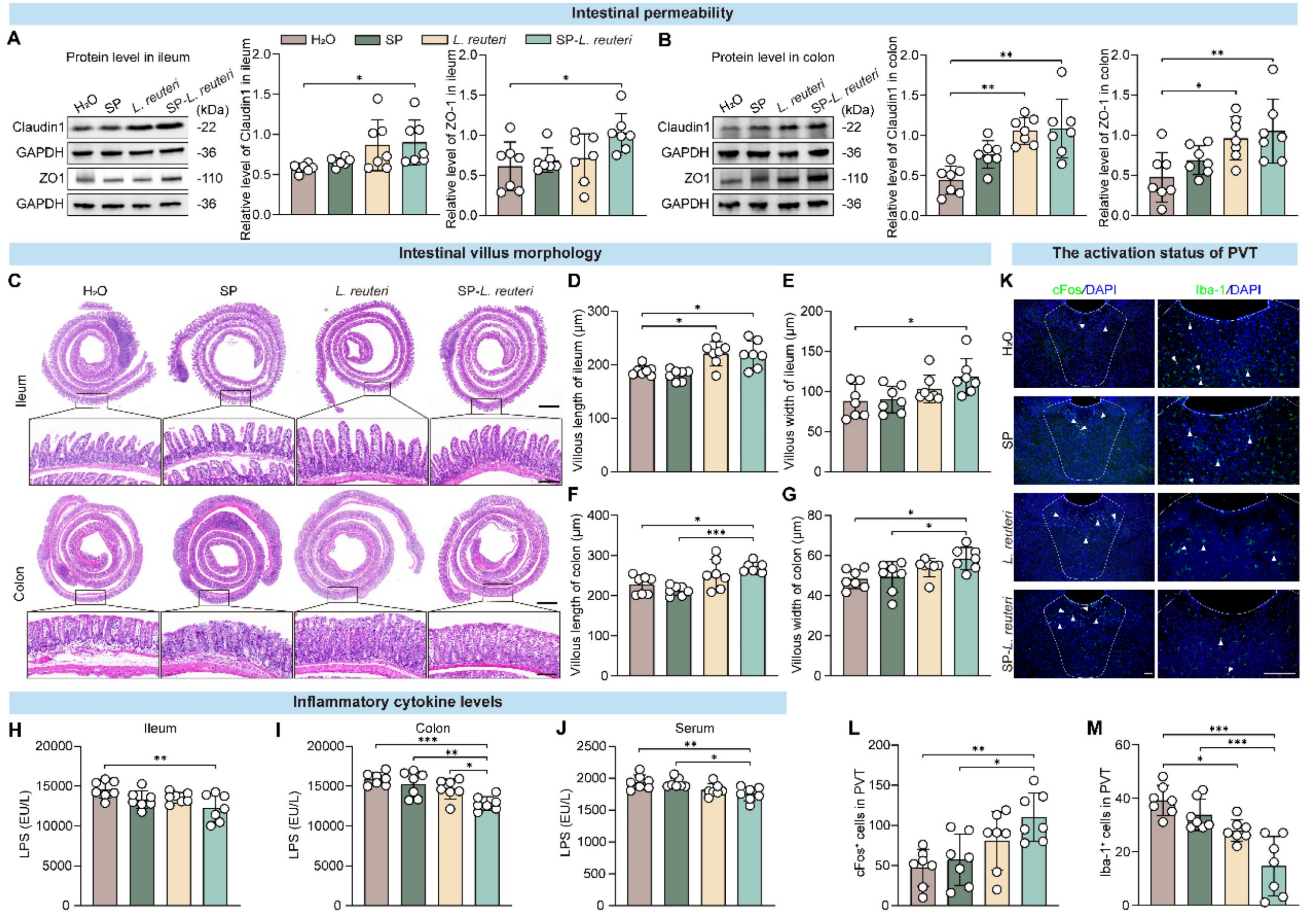
** SP-*L. reuteri* enhances intestinal barrier function and reduces the inflammatory response in the PVT in BTBR mice.** A-B, Protein expression of tight junction proteins (Claudin-1 and ZO-1) in (A) ileum and (B) colon tissues. C-G, Representative graphs of (C) HE staining, (D, F) villus length and (E, G) width data for the ileum and colon. Scale bar = 500 µm; the following picture is the enlargement of the region indicated by the black rectangle from the above picture. Scale bar = 50 µm. H-J, LPS levels in the (H) ileum, (I) colon, and (J) serum. K-M, (K) Immunofluorescence images and statistics of the number of (L) c-fos- and (M) Iba-1-positive cells (White arrow) in the PVT brain region. Scale bar = 100 µm. The error bars represent the standard deviations (n = 7). Statistical significance was assessed via one-way ANOVA (* *p* < 0.05, ** *p* < 0.01, *** *p* < 0.001).

The gut microbiota plays an important role in the occurrence and progression of ASD via the microbiota‒gut‒brain axis 33. Altered gut microbiota and disruption of intestinal barrier function are observed in mouse models of ASD 34. Our findings revealed that, compared with C57BL/6J mice, BTBR mice presented increased intestinal permeability (Figure S11A-B). Changes in intestinal permeability may lead to molecules in the intestinal cavity crossing the epithelial barrier, which can result in “leaky gut syndrome” 35. We further observed the intestinal morphology and detected the expression of intestinal barrier-associated proteins. After SP-*L. reuteri* and *L. reuteri* intervention, the expression of tight junction proteins was significantly increased in BTBR mice (Figure 7A-B), and both the length and width of villi in the small intestine and large intestine were also significantly increased (Figure 7C-G). The intestinal barrier and structure were more complete in the SP-*L. reuteri*-treated group than in the other groups. The levels of cytokines in the intestinal tissue and serum were also detected. SP-*L. reuteri* treatment significantly reduced the level of lipopolysaccharide (LPS) in the ileum, colon and serum of BTBR mice (Figure 7H-J). There were no significant differences in the levels of inflammatory factors (IL-6, TNF-α) and oxidative stress markers (MDA) among the individual groups (Figure S11C-E).

Gut microbes are a key source of LPS 36, 37, and there is an association between LPS secretion and gut microbiome disturbance 38. Increased intestinal permeability allows the hyper-translocation of microbiota-derived endotoxins, such as LPS, into the blood circulation, which is consistent with our findings (Figure 7H-J). Peripheral LPS can activate microglia and then cause neuroinflammation 39, and excessive microglial activation in multiple brain regions in young adult subjects with ASD 40. Therefore, we further examined neural activation in the PVT (paraventricular thalamic nucleus), an ASD-related brain region 41, 42. Immunostaining of c-fos revealed that SP-*L. reuteri* treatment significantly activated the neurons in the PVT, suggesting that SP-*L. reuteri* has a regulatory effect on the PVT (Figure 7K-L). Additionally, the number of ionized calcium binding adaptor molecule 1 (Iba1)-positive cells, which are activated microglia, was significantly reduced in the SP-*L. reuteri* group (Figure 7K, M), reflecting the attenuation of the neuroinflammatory response in the PVT.

In summary, these data suggested that SP-*L. reuteri* reduces the production of intestinal LPS, enhances intestinal integrity, prevents toxicants such as LPS from entering the blood from the intestine, reduces the activation of neuroinflammation in the PVT, and thereby improves social deficits.

## Discussion and conclusion

Exogenous probiotic supplementation can effectively alleviate the symptoms of several psychiatric disorders, including ASD. However, due to the influence of the gastrointestinal environment, probiotics cannot effectively colonize host intestines, which limits their oral bioavailability. In the present study, we developed a novel oral delivery system for intestinal bacteria, SP-assisted intestinal bacteria, using natural SP as a microcarrier. For multiple intestinal bacteria with different shapes and gram-staining properties, the SP-intestinal bacteria system has a universally longer intestinal residence time and greater colonization efficiency than unmodified intestinal bacteria. In addition, SP-*L. reuteri* enhances the therapeutic efficacy of *L. reuteri* in alleviating social impairment in ASD model mice by restructuring the gut microbiota, reducing LPS entry into the blood and mediating the neuroinflammatory response in the PVT region.

As a helical microalga composed of multiple cells, SP has natural aqueous channels and junctional pores on the cell wall for transmembrane exchange and slime secretion 43, 44. These natural channels allow small molecules to diffuse into SP cells under the force of osmotic pressure 45, 46. Therefore, SP has great potential for targeted drug delivery both *in vivo* and *in vitro* 47. The pore size of SP is 14-16 nm, while the diameter of bacteria is mostly 0.5-5 μm. Bacteria cannot enter pores but can attach to the surface of SP. At present, SP are mainly used for the loading of small-molecule chemical drugs. To our knowledge, we developed the first delivery system using SP as a natural microcarrier for intestinal bacteria and confirmed its safety and intestinal colonization ability for multiple bacterial strains.

Bacteria adhere to other biological surfaces through adsorption and subsequent formation of microcolonies and biofilms 20. First, adhesive factors on the bacterial surface, such as pili, capsules, and extracellular polysaccharides, play crucial roles 19, 20. These adhesive factors can interact with specific sites or molecules on the surface of SP, enabling the adhesion of bacteria to SP. For example, pili can help bacteria move forwards and adhere to the surface of SP through wiggling movements. Second, environmental factors can also affect bacterial adhesion. Factors such as pH, temperature, and salt concentration can influence the charge on the bacterial surface and the properties of the SP surface, thereby affecting the adhesion between them. Additionally, the adhesion between bacteria and SP may involve interactions with other biological molecules, such as proteins and polysaccharides 48. These molecules may form bridges between the bacterial surface and the SP surface, further promoting their adhesion. SP has a large surface area and adsorption capacity 49, which may promote the adhesion of bacteria to the surface of SP.

SP is a dietary supplement with a variety of biological activities and is cultivated in large quantities. Many of its varieties have been commercialized as nutraceutical supplements, demonstrating their utility and high biosafety as oral pharmaceutical formulations. The construction of SP-intestinal bacteria requires no other toxic additives and is a simple, safe, effective, and low-cost approach. Moreover, intestinal bacteria attached to the surface of SP can closely contact the surface of intestinal villi and subsequently interact with the host. The SP body can also be loaded with a variety of small-molecule drugs for disease treatment 50. Therefore, this delivery system can achieve multiple treatment effects and enhance therapeutic efficacy in the future.

As a microcarrier that is 200-500 μm in size, SP showed preferential retention between the intestinal villi (300-600 μm) and progressive degradation in the intestinal tract. These characteristics enable the comprehensive and significant distribution of SP throughout the small intestine 14. The results of the present study confirmed the high gut adhesion efficiency of SP-intestinal bacteria in many respects, and most of the bacteria still adhered to the surface of the SP in the intestine, which was confirmed by the SEM image of the SP-*E. coli* in the SIFs (Figure S1E). Therefore, we speculate that after SP-bacteria adhere to the intestine, the SP will gradually degrade in the intestinal environment 16. The intestinal bacteria that adhere to the SP surface after SP degradation will continue to remain in the intestine, leading to the long-term retention of bacteria in the intestines. However, elucidating the specific details will require further investigation.

ASD is a complex neurodevelopmental disorder whose origins are influenced by genetic and environmental factors 51. Multiple studies have shown that intestinal microbial dysbiosis is associated with the development of autism 34, 52. Therefore, probiotic supplementation through the regulation of the microbiota-gut-brain axis has gained increasing attention in the treatment of ASD. Supplementing BTBR mice with *L. reuteri* has been found to alleviate their social ability and social novelty 53, and other studies also indicate that it can effectively rescue their social ability and social novelty, but no changes in repetitive behaviour or sociability were observed 54. In *shank3^-/-^* mice, some probiotics not only restore social behaviour but also eliminate repetitive behaviour 55. The reasons for these differences in therapeutic efficacy may be related to factors such as differences in the autism model mice, the specificity of probiotic strains, and engraftment efficiency. The actual number of probiotics colonizing the gastrointestinal tract has a substantial impact on their therapeutic efficacy. Our work demonstrated that SP-*L. reuteri* has a robust therapeutic effect on ASD, as it ameliorates sociability and social novelty. The SP-*L. reuteri* system was more effective at improving the core symptoms of ASD than *L. reuteri*.

Microbiota diversity serves as a metric to assess species diversity within a particular region or habitat. However, we found controversial findings in children with ASD 56, including reduced gut microbiota diversity 57, as well as increased 58 and unaltered diversities 59, 60 compared with those of typically developing children. Inconsistent gut microbiota diversities were also observed among different ASD mouse models 5, 6, 53, 58, 61, 62. This may be related to various factors, such as different regions, sexes, and dietary environments. When specific changes in the intestinal environment are evaluated, in addition to considering microbial diversity, more detailed metrics, such as species relative abundance, are needed for a comprehensive assessment to reflect the structure and characteristics of the gut microbiota community more fully.

Compared to that of wild-type mice, the gut microbial composition of ASD model mice was significantly altered, with a decrease in *L. reuteri* abundance.* L. reuteri* supplementation corrects social deficits in ASD model mice by altering the gut microbial composition but has no significant effect on the overall microbial composition of the host 63, 64. At the phylum level, the F/B ratio is decreased in autism model mice. At the genus level, the abundance of *Lactobacillus* is decreased, and the abundance of *Lachnospiracea_incertae_sedis* is increased in autistic children 65. As in previous studies, *L. reuteri* supplementation in this study did not significantly affect the overall gut microbial structure or diversity. However, the gut microbial structure of mice supplemented with SP-*L. reuteri* significantly differed from that of BTBR- and *L. reuteri*-treated mice, and the α-diversity was reduced. After treatment with SP-*L. reuteri*, the F/B ratio increased, the relative abundances of *Ruminococcaceae*, *Lachnospiraceae*, and *Mucispirillum* decreased, and the relative abundances of* Lactobacillus* and* Alloprevotella* increased. *Ruminococcaceae* and *Mucispirillum* are associated with inflammation 63, 64 and have considerable pathogenic potential. *Alloprevotella* is thought to be a beneficial bacterium that produces butyrate and promotes an anti-inflammatory environment 66.

Research shows that autistic females may exhibit lower levels of circumscribed interests and repetitive behaviours than their male counterparts 67. Animal experiments have confirmed that the effects of probiotics in the treatment of autism are sex dependent 55. Since male mice are more likely to exhibit autism phenotypes than female mice are, only male mice were used in this study, and the therapeutic effect on female mice needs to be further explored in subsequent experiments.

This study has several limitations. First, we improved the efficiency of intestinal colonization and enhanced the therapeutic effect of ASD treatment by developing an SP-intestinal bacterial delivery system. However, the sustained therapeutic effects of SP-*L. reuteri* treatment have not been investigated. Second, the present study used only BTBR mice as an ASD model; further studies are needed to verify the efficacy of the formulation in other autism or psychiatric models. Finally, the experimental data were all derived from mice, and these findings need to be validated in patients with ASD in the future to accelerate their application in the clinic.

In summary, to improve the therapeutic efficacy of beneficial intestinal bacteria in treating mental illness, for the first time, we used SP as a microcarrier of beneficial intestinal bacteria to construct an SP-assisted delivery system, SP-intestinal bacteria, which significantly improved the survival rate of bacteria in the gastrointestinal tract. The SP-intestinal bacterial delivery system is a general strategy that results in high intestinal colonization efficiency and retention capacity both *in vivo* and *in vitro*. Moreover, treatment with SP-intestinal bacteria significantly alleviated the core symptoms of ASD, demonstrating a powerful therapeutic effect. This study provides a new direction for the promotion and application of microbial therapy and new options for gut- and gut-brain-associated disease treatment.

## Supplementary Material

Supplementary figures and tables.

## Figures and Tables

**Table 1:** Optimal culture conditions for SP-intestinal bacteria (10 mL)

Bacterium shape	Gram Staining	Strain	Incubation time	SP content	OD_600_	Rotating speed	Number loaded
rod-shaped	gram-negative	*E. coli*	4 h	0.8 g	0.8	110 rpm	3.62×10^8^/mL
	gram-positive	*L. reuteri*	4 h	0.8 g	0.8	110 rpm	7.04×10^8^/mL
spherical	gram-positive	*S. thermophilus*	4 h	0.6 g	1	60 rpm	7.28×10^5^/mL
	gram-negative	*B. ovatus*	4 h	0.8 g	0.8	110 rpm	6.65×10^8^/mL

## Abbreviations

ASD: Autism spectrum disorder
SP: *Spirulina platensis*
SP-intestinal bacteria: SP-assisted intestinal bacteria delivery system
*L. reuteri*: *Lactobacillus reuteri*
*E. coli*: *Escherichia coli*
*S. thermophilus*: *Streptococcus thermophilus*
*B. ovatus*: *Bacteroides ovatus*
F/B ratio: Firmicutes/Bacteroidetes ratio
TNF-α: Tumor necrosis factor-alpha
IL-6: Interleukin 6
MDA: Malondialdehyde
LPS: Lipopolysaccharide
PVT: Paraventricular thalamic nucleus
SGF: Simulated gastric fluid
SIF: Simulated intestinal fluid
BS: Bile salts
SEM: Scanning electron microscopy
PCoA: Principal coordinate analysis
LDA: linear discriminant analysis effect size
LEfSe: LDA effect size
