# Supplementary material for: Microalgae-based bacteria for oral treatment of ASD through enhanced intestinal colonization and homeostasis
Source: Theranostics. 2025 Jan 13;15(6):2139–58. doi: 10.7150/thno.103737 (PMC11840722; doi:10.7150/thno.103737)
Supplement: Supplementary file 1 — Supplementary figures and tables. [file thnov15p2139s1.pdf]

Supporting Information

Microalgae-based bacteria for oral treatment of ASD through enhanced intestinal colonization and homeostasis

Rongrong Yang, Li Ma, Huan Peng, Yifang Zhai, Gengyao Zhou, Linjuan Zhang, Lixia Zhuo, Wei Wu, Yuxi Guo, Jiao Han, Linlin Jing, Xinyu Zhou, Xiancang Ma, Yan Li

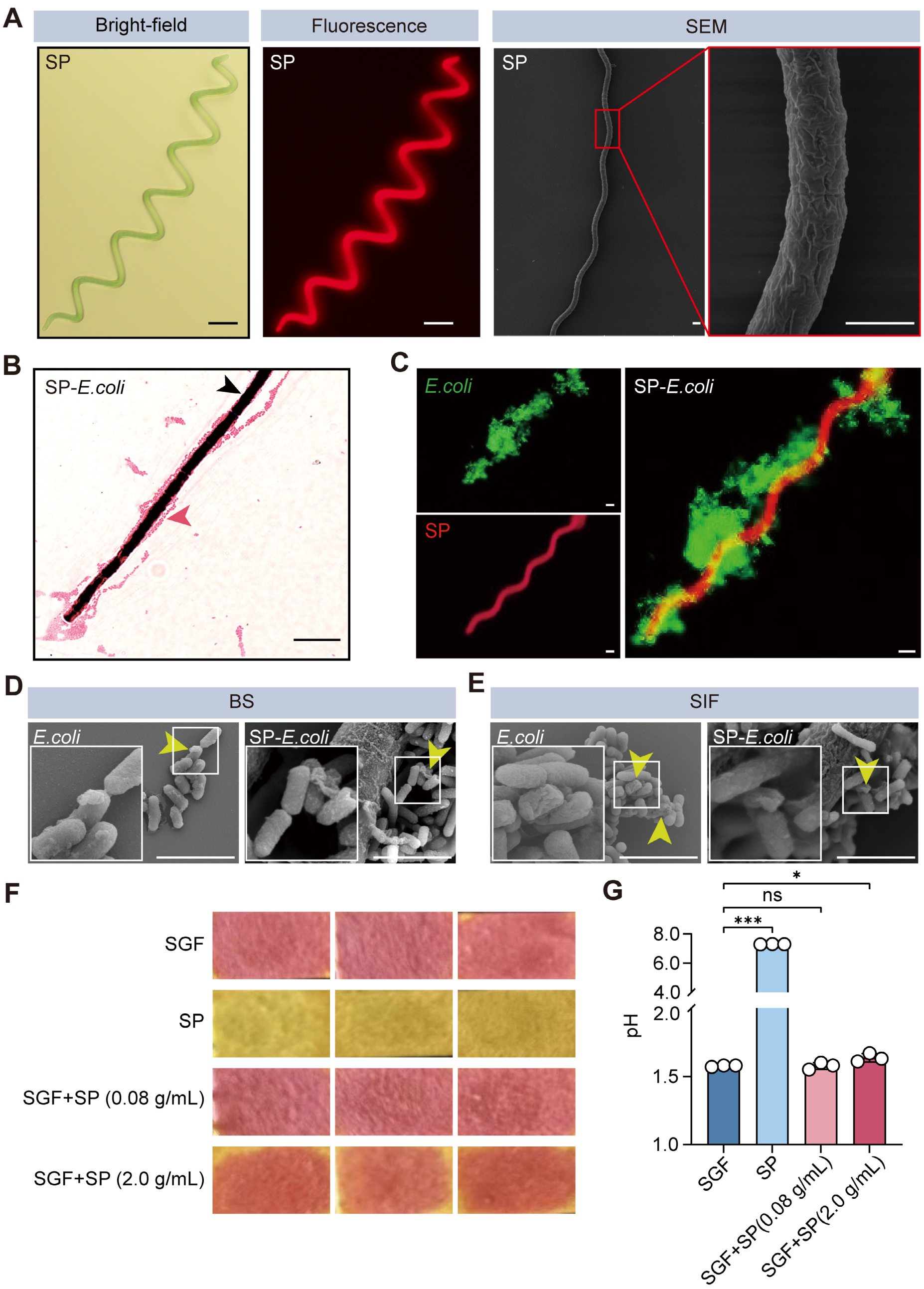

Figure S1. The identification of SP-*E. coli*. A, Bright field microscopy, fluorescence microscopy, and SEM images of SP. B-C, Representative image of (B) Gram staining and (C) fluorescence microscopy of SP-*E. coli*. D-E, SEM images of *E. coli* and SP-*E. coli* after exposure to (D) BS and (E) SIF. Scale bar = 10 μm. F-G, pH values representative images and statistics of SGF, SP, SGF supplemented with SP (0.08 g/mL, 2 g/mL). Statistical significance was assessed using one-way ANOVA (ns: not significant, * $p < 0.05$, *** $p < 0.001$).

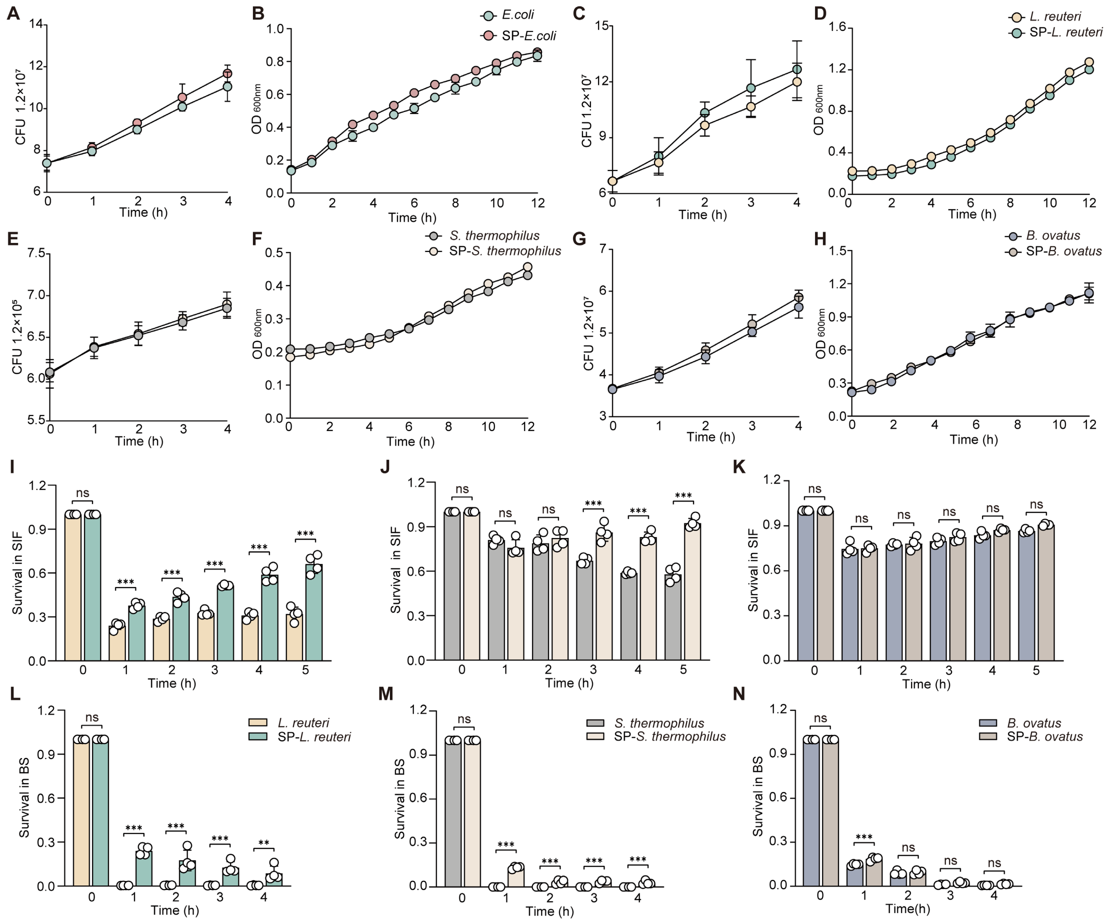

Figure S2. Bacterial survival rate and intestinal tolerance testing. A-H, (A, C, E, G) The bacterial count was measured via plate counting. (B, D, F, H) Growth curve in LB medium. The OD_{600} was recorded at 30-min intervals using a microplate reader. (A, B) *E. coli* and SP-*E. coli*, (C, D) *L. reuteri* and SP-*L. reuteri*, (E, F) *S. thermophilus* and SP-*S. thermophilus,* (G, H) *B. ovatus* and SP-*B. ovatus*. I-N, Bacterial survival after exposure to (I, J, K) SIF and (L, M, N) simulated bile salts. (I, L) *L. reuteri* and SP-*L. reuteri*, (J, M) *S. thermophilus* and SP-*S. thermophilus*, (K, N) *B. ovatus* and SP-*B. ovatus*. The data are expressed as the mean ± SD (n = 4 per group). Statistical significance was assessed using two-way ANOVA (ns: not significant, ** $p < 0.01$, *** $p < 0.001$).

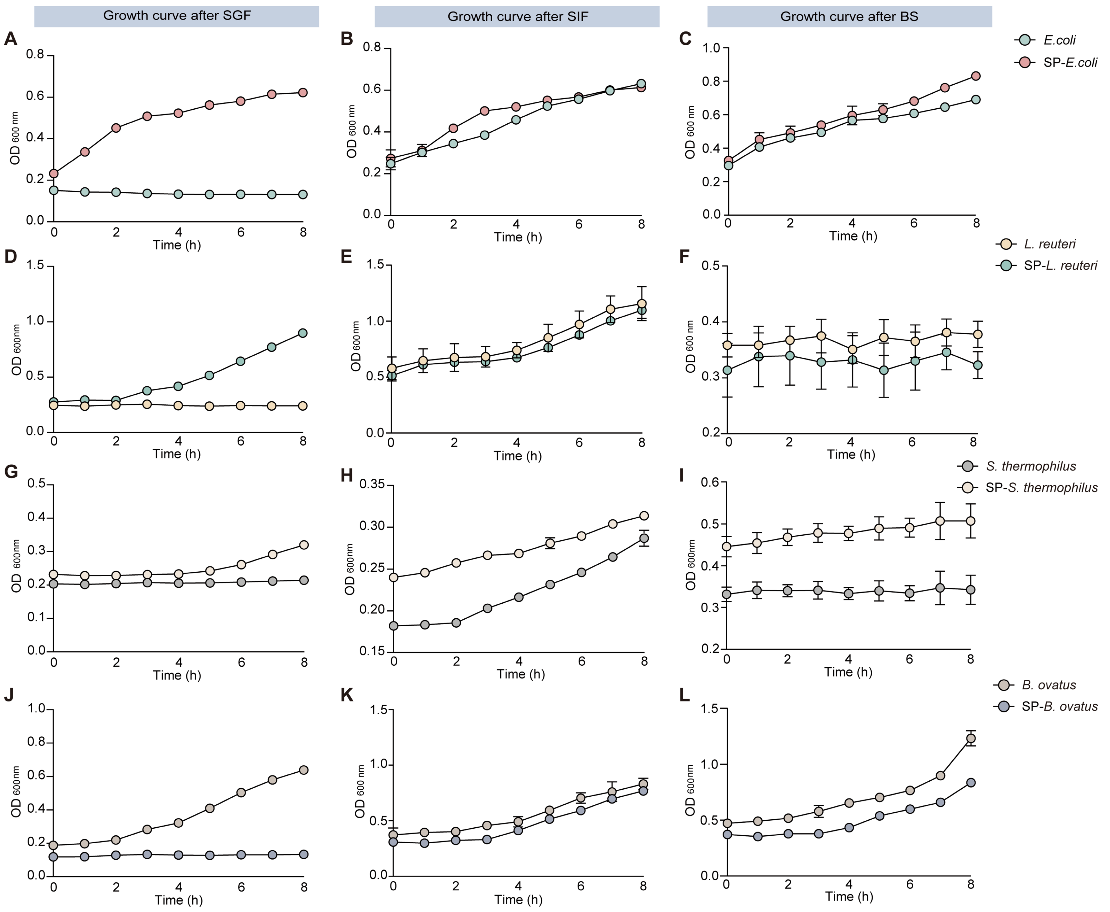

Figure S3. Growth curve determination. Growth curves in culture medium after 15 min of incubation in (A, D, G, J) SGF and after 1 h of incubation in (B, E, H, K) SIF and (C, F, I, L) BS. The OD_{600} was recorded at 1 h intervals using a microplate reader. (A, B, C) *E. coli* and SP-*E. coli*, (D, E, F) *L. reuteri* and SP-*L. reuteri*, (G, H, I) *S. thermophilus* and SP-*S. thermophilus*, (J, K, L) *B. ovatus* and SP-*B. ovatus*.

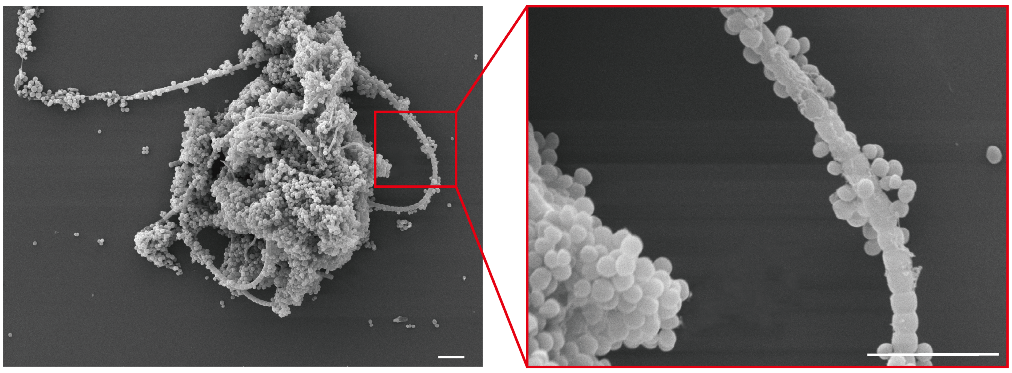

Figure S4. SEM image of *Nostoc* sp. - *B. ovatus*. Scale bar = 10 µm.

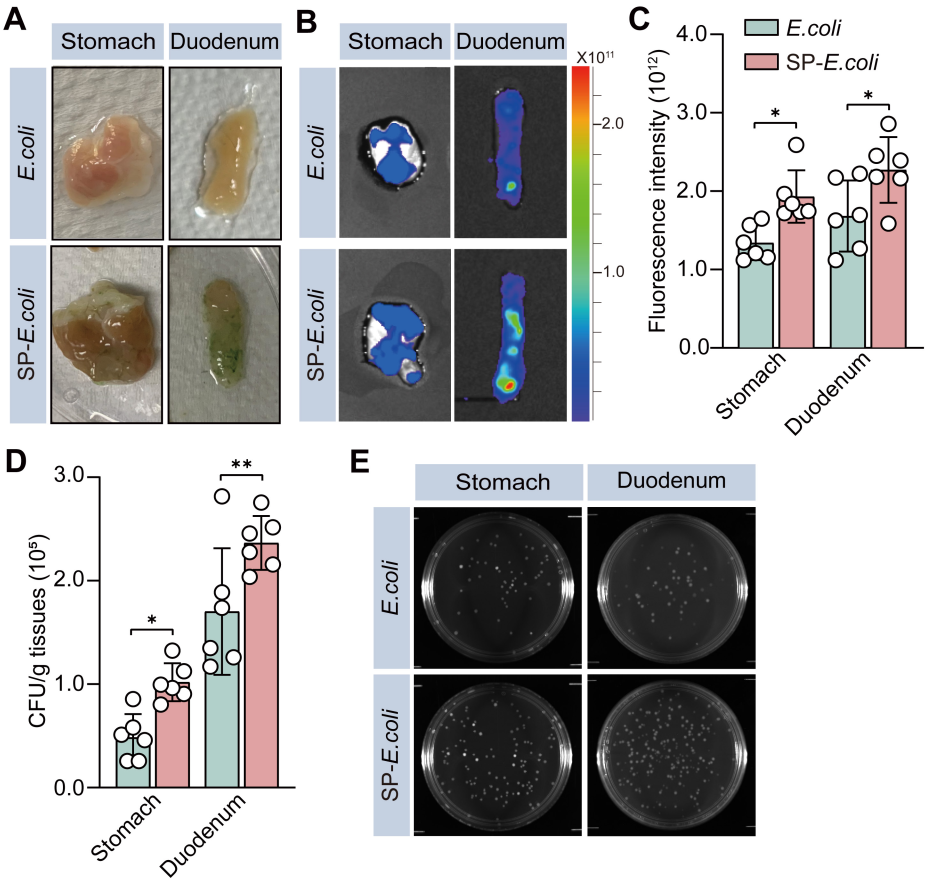

Figure S5. The adhesion of *E. coli* and SP-*E. coli* in the stomach and intestine. A-E, (A) Digital images, (B) fluorescence imaging images and (C) corresponding statistical analysis of fluorescence intensity, (D) statistical analysis and (E) LB agar plates for colony counting of stomach and duodenum incubated with *E. coli* or SP-*E. coli*. The mouse intestine was removed and separated into intestinal segments with a length of 1.5 cm, after which the contents were removed. An equal amount of 150 μL of *E. coli* or SP-*E. coli* was injected into the intestinal lumen, and the intestinal tissue was ligated at both ends. After incubation for 30 min, the samples were rinsed to completely remove unattached bacteria. The data are expressed as the mean ± SD. (n = 6 per group). Statistical significance was assessed using two-way ANOVA. * $p < 0.05$, ** $p < 0.01$.

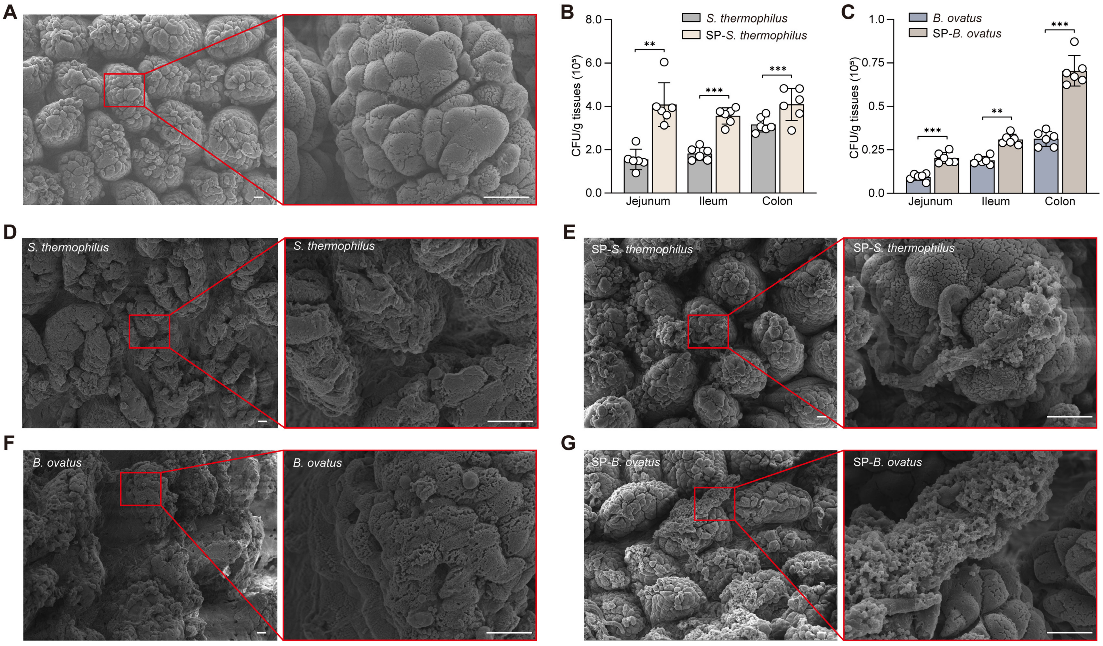

Figure S6. Scanning electron microscopy images and bacterial count statistics. A, SEM image of intestinal tissues in control group. The ileum tissues of mice were obtained, rinsed three times with 0.9% saline to eliminate all contents, and then exposed to 0.9% saline for 30 min. B-C, The number of bacteria adhering to different intestinal tissues. (B) *S. thermophilus*, and SP-*S. thermophilus*, (C) *B. ovatus* and SP-*B. ovatus*. D-G, SEM image of (D) *S. thermophilus*, and (E) SP-*S. thermophilus,* (F) *B. ovatus* and (G) SP-*B. ovatus*. Scale bar = 10 µm. The data are expressed as the mean ± SD. (n = 6 per group). Statistical significance was assessed using two-way ANOVA (ns: not significant, ** $p < 0.01$, *** $p < 0.001$).

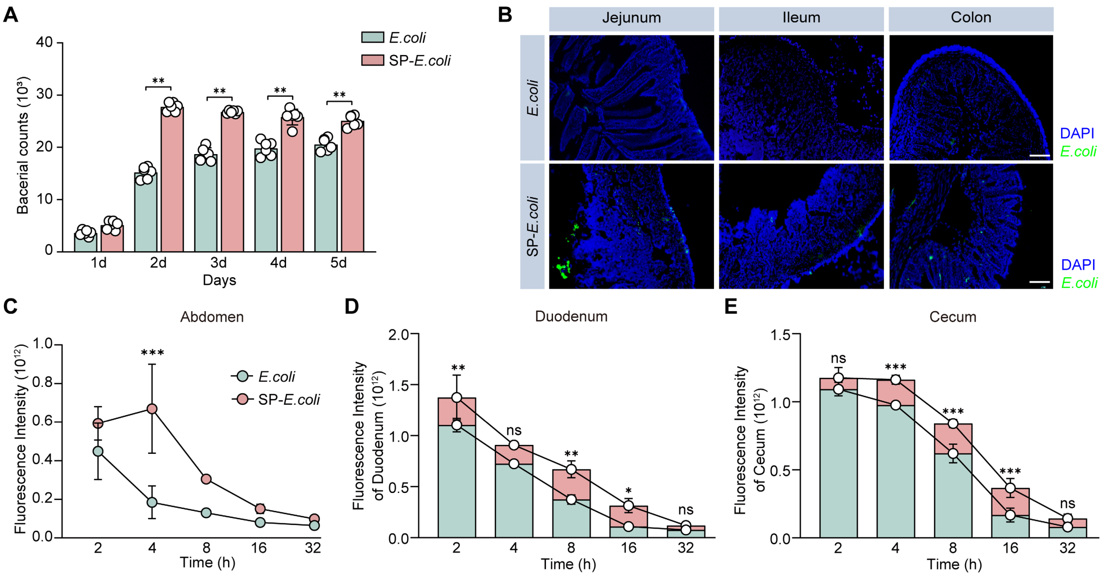

Figure S7. Analysis of colony counts in fecal samples and fluorescence observation of intestinal tissues. A, Bacterial counts of fecal samples from C57BL/6J mice. Fecal samples were collected after gavage with *E. coli* and SP-*E. coli* for different durations and homogenized for plate counting. B, Fluorescence microscopy images of intestinal tissue (jejunum, ileum, and colon). Scale bar = 100 μm. The data are expressed as the mean ± SD (n = 6 per group). C-E, Fluorescence intensities in the (C) abdominal area of mice and (D) duodenum and (E) cecum sampled at different periods after oral gavage of 3×10^{8} CFU of *E. coli* and SP-*E. coli*. Statistical significance was assessed using two-way ANOVA (ns: not significant, * $p < 0.05$, ** $p < 0.01$, *** $p < 0.001$).

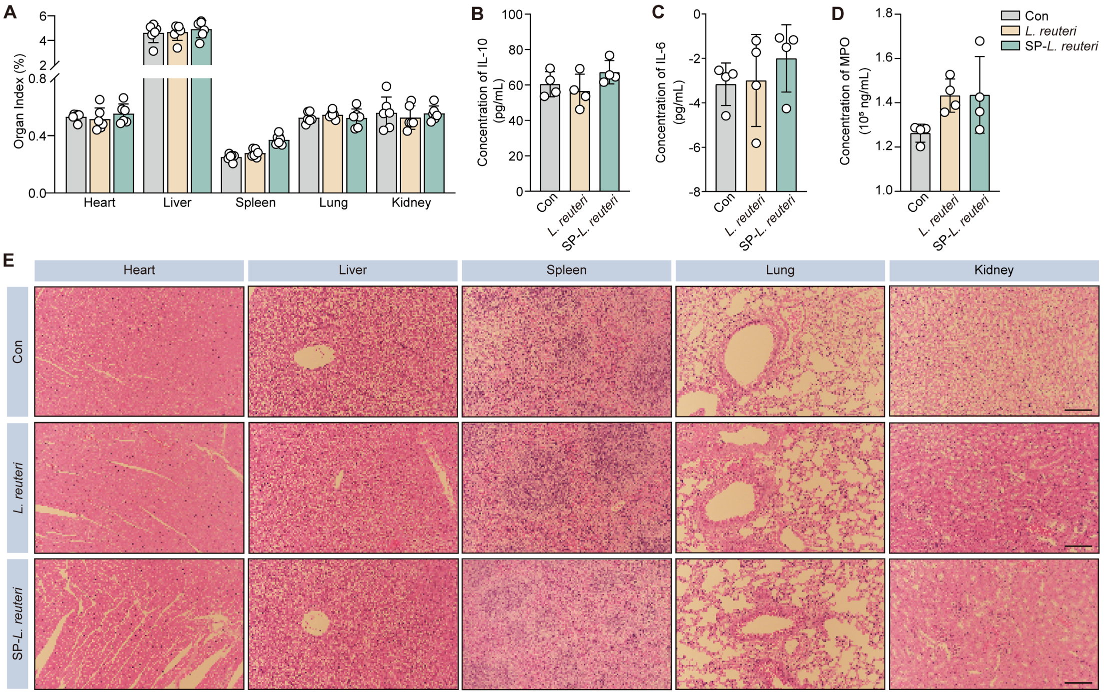

Figure S8. Oral biosafety in mice. A, Mouse organ indices. Organ index = organ weight (mg)/mouse body weight (g). B-D, The levels of (B) IL-10, (C) IL-6, and (D) MPO in serum. E, Representative images of HE-stained mouse organs (including the heart, liver, spleen, lungs, and kidney). After 14 days of intragastric administration, the mice were euthanized, and blood and organs were collected for further analysis. The data are expressed as the mean ± SD (n = 4 per group). The significance of differences was determined using two-way ANOVA or one-way ANOVA.

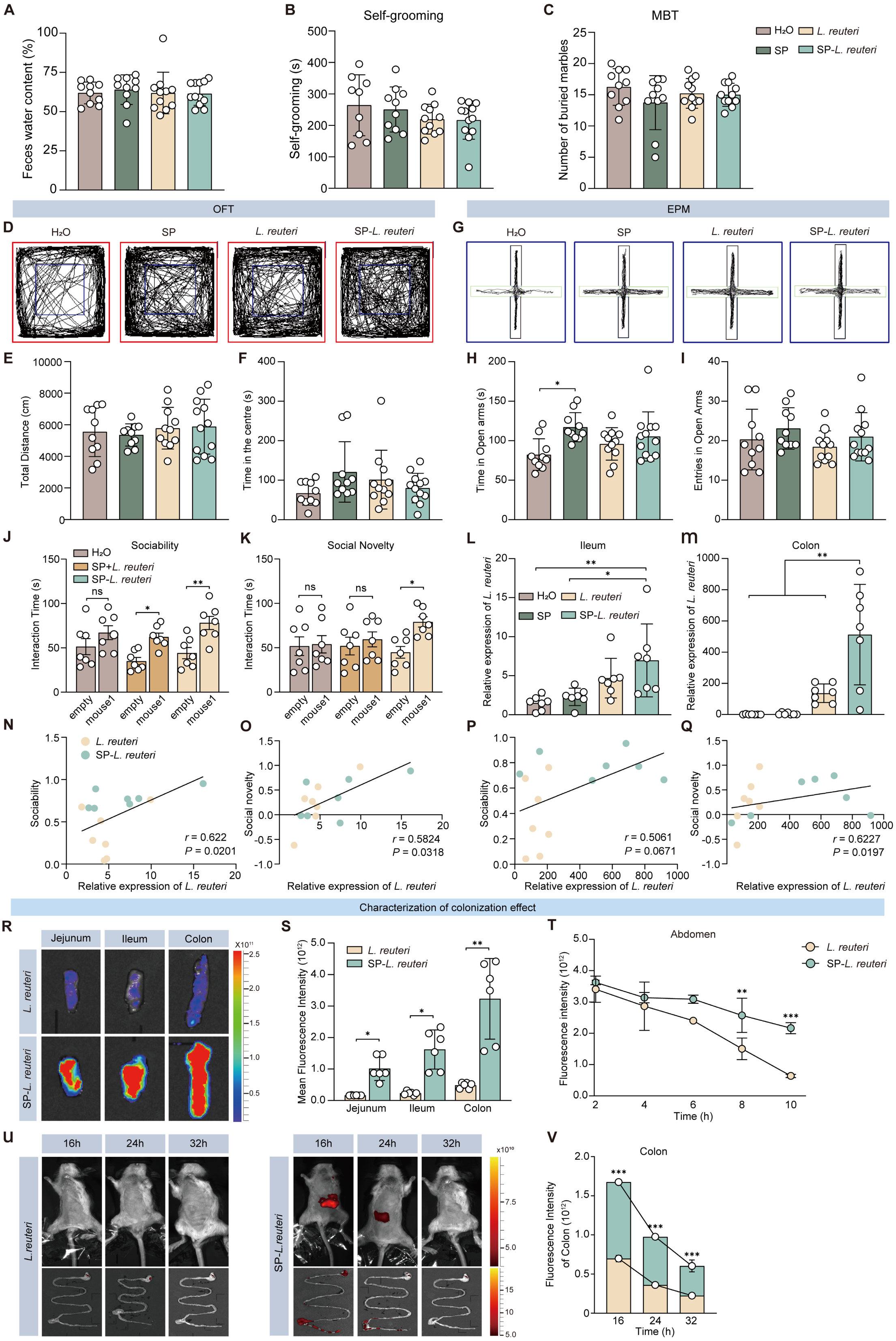

Figure S9. ***SP-L. reuteri*** **colonization efficiency in the intestine and its impact on anxiety behavior in BTBR mice.** A, Feces water content in different treatment groups. B, Self-grooming behavior was assessed by quantifying the total time spent grooming over 30 min. C, The number

of buried marbles in each group. D-I, Anxiety-like behavior was assessed by the (D-F) open field test (OFT) and (G-I) elevated plus maze (EPM) test. (D) Trajectory diagram, (E) total distance moved, and (F) time spent in the central area of the OFT. (G) Movement trajectory diagram, (H) total time spent in the open arms and (I) the number of open arm entries in the EPM test. The data are expressed as the mean ± SD (n = 10-12 per group). J-K, Statistics on the interaction time of (J) sociability and (K) social novelty of mice with various treatments. The data are expressed as the mean ± SDs (n = 7 per group). L-M, Relative expression of *L. reuteri* in the (L) ileum and (M) colon. N-Q, Correlation analysis between the expression of (N, O) the ileum *L. reuteri* and (P, Q) the colon *L. reuteri* and (N, P) sociability and (O, Q) social novelty. Spearman's rank correlation analysis was used for linear correlation analysis. (n = 7 per group). R-S, (R) Representative fluorescence images and (S) corresponding statistical analysis of fluorescence intensity. The data are expressed as the mean ± SD (n = 6 per group). T, Fluorescence intensities in the abdominal area of mice at different periods after oral gavage of 3×10^8 CFU of *L. reuteri* or SP-*L. reuteri*. U-V, (U) Representative live animal fluorescence images and (V) fluorescence intensities of colon sampled at 16 h, 24 h, 32 h after oral gavage of 3×10^8 CFU of *L. reuteri* or SP-*L. reuteri.* The significance of differences was determined using two-way ANOVA or one-way ANOVA. (ns: not significant, * $p < 0.05$, ** $p < 0.01$, *** $p < 0.001$).

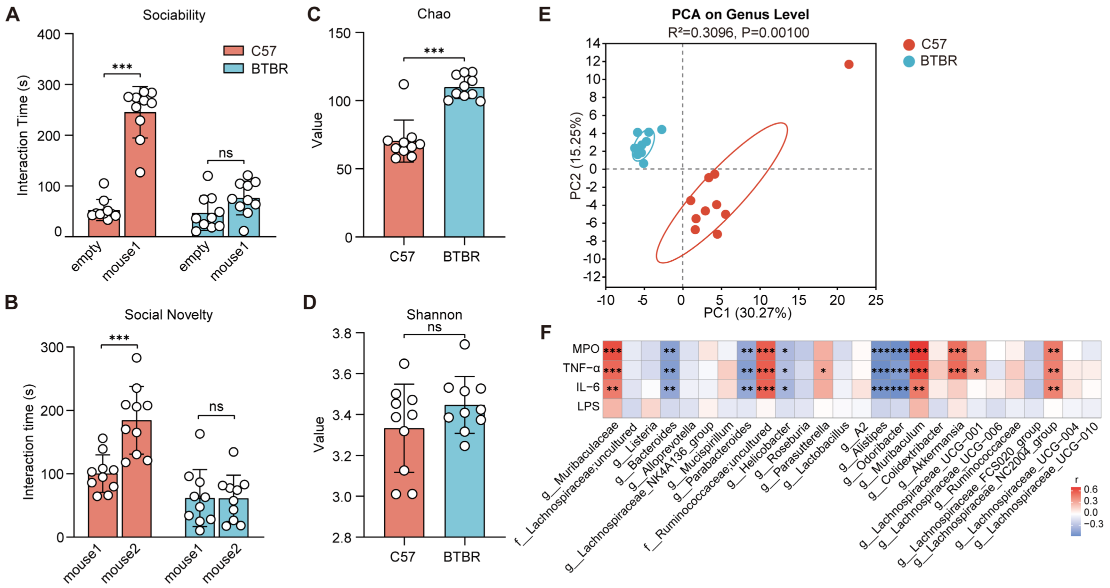

Figure S10. Differences in the behaviour and intestinal microbiota between C57BL/6J and BTBR mice. A-B, The social behaviour of C57BL/6J and BTBR mice was assessed via the 3-chamber test (n = 10). (A) Interaction time of sociability and (B) social novelty. C-D, α-Diversity of the gut microbiota of the mice. (C) Chao indices reflect the richness of each sample sequence, and (D) Shannon indices reflect the diversity of the intestinal microbial composition. E, Principal component analysis (PCA) of the mouse microbiota community structure. F, Heat map of the Spearman correlation between the top 20 gut microbiota constituents and immune factors. The red and blue dots represent positive and negative correlations, respectively. The significance of differences was determined using two-way ANOVA or Student's two-tailed t-test. (ns: not significant, * $p < 0.05$, ** $p < 0.01$, *** $p < 0.001$).

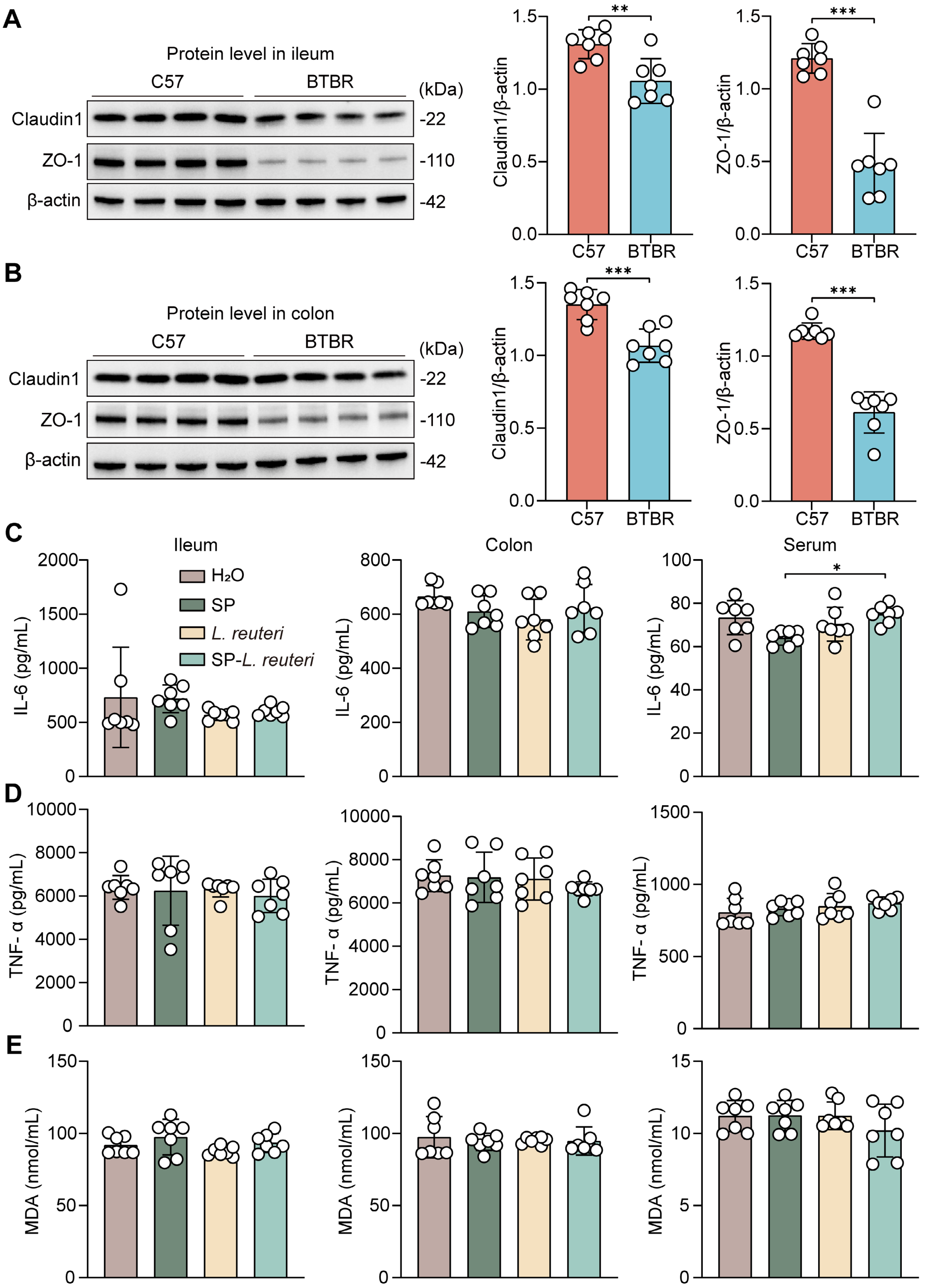

Figure S11. Expression of tight junction-associated proteins and cytokines in the intestine. A-B, Expression of tight junction proteins (Claudin-1 and ZO-1) in (A) ileum and (B) colon tissues of C57BL/6J and BTBR mice. C-E, (C) IL-6, (D) TNF-α, and (E) MDA levels in the ileum, colon, and serum. The data are expressed as the mean ± SD (n = 7). Statistical significance was assessed via unpaired t tests and one-way ANOVA (* $p < 0.05$, ** $p < 0.01$, *** $p < 0.001$).

Table S1. Orthogonal experimental design of SP-intestinal bacteria

ID	Incubation time	SP content	OD_{600}	Rotating speed	Number of loaded *E. coli*	Number of loaded *L. reuteri*
1	2 h	0.2 g	0.2	60 rpm	9.6×10^6/mL	2.4×10^6/mL
2	2 h	0.4 g	0.4	110 rpm	1.8×10^7/mL	0.8×10^6/mL
3	2 h	0.6 g	0.8	160 rpm	1.28×10^7/mL	-
4	2 h	0.8 g	1	220 rpm	8.8×10^6/mL	-
5	4 h	0.2 g	0.4	160 rpm	9.6×10^6/mL	-
6	4 h	0.4 g	0.2	220 rpm	2.32×10^7/mL	-
7	4 h	0.6 g	1	60 rpm	2.16×10^7/mL	-
8	4 h	0.8 g	0.8	110 rpm	3.62×10^8/mL	7.04×10^8/mL
9	6 h	0.2 g	0.8	220 rpm	1.22×10^8/mL	-
10	6 h	0.4 g	1	160 rpm	6.4×10^6/mL	1.04×10^7/mL
11	6 h	0.6 g	0.2	110 rpm	1.44×10^7/mL	1.6×10^6/mL
12	6 h	0.8 g	0.4	60 rpm	7.2×10^6/mL	9.6×10^6/mL
13	8 h	0.2 g	1	110 rpm	1.29×10^8/mL	7.2×10^6/mL
14	8 h	0.4 g	0.8	60 rpm	8.4×10^7/mL	2.64×10^7/mL
15	8 h	0.6 g	0.4	220 rpm	9.6×10^7/mL	1.6×10^7/mL
16	8 h	0.8 g	0.2	160 rpm	8.8×10^6/mL	3.2×10^6/mL

ID	Incubation time	SP content	OD_{600}	Rotating speed	Number of loaded *S. thermophilus*	Number of loaded *B. ovatus*
1	2 h	0.2 g	0.2	60 rpm	3.04×10^5/mL	0.8×10^6/mL
2	2 h	0.4 g	0.4	110 rpm	2.16×10^5/mL	2.35×10^8/mL
3	2 h	0.6 g	0.8	160 rpm	3.2×10^4/mL	0.8×10^6/mL
4	2 h	0.8 g	1	220 rpm	6.4×10^4/mL	3.2×10^6/mL
5	4 h	0.2 g	0.4	160 rpm	-	-
6	4 h	0.4 g	0.2	220 rpm	-	1.52×10^7/mL
7	4 h	0.6 g	1	60 rpm	7.28×10^5/mL	-
8	4 h	0.8 g	0.8	110 rpm	1.28×10^5/mL	6.65×10^8/mL
9	6 h	0.2 g	0.8	220 rpm	-	1.31×10^8/mL
10	6 h	0.4 g	1	160 rpm	-	0.8×10^6/mL
11	6 h	0.6 g	0.2	110 rpm	-	4.56×10^8/mL
12	6 h	0.8 g	0.4	60 rpm	4.8×10^5/mL	2.38×10^8/mL
13	8 h	0.2 g	1	110 rpm	-	-
14	8 h	0.4 g	0.8	60 rpm	2.72×10^5/mL	-
15	8 h	0.6 g	0.4	220 rpm	2.187×10^5/mL	2.4×10^7/mL
16	8 h	0.8 g	0.2	160 rpm	-	1.68×10^7/mL

- indicates that the number is very small

Table S2. List of qPCR primer sequences

Name	Sequence (5'→3')
16s F	GAGTTTGATCCTGGCTCAG
16s R	GGTTACCTTGTTACGACTT
Lactobacillus reuteri F	TGGCTTTGGCTATCACTCTGG
Lactobacillus reuteri R	AGATTCCCTACTGCTGCCTCC
